# A 30-Year Study of Impacts, Recovery, and Development of Critical Effect Sizes for Endocrine Disruption in White Sucker (*Catostomus commersonii*) Exposed to Bleached-Kraft Pulp Mill Effluent at Jackfish Bay, Ontario, Canada

**DOI:** 10.3389/fendo.2021.664157

**Published:** 2021-04-22

**Authors:** Erin J. Ussery, Mark E. McMaster, Mark R. Servos, David H. Miller, Kelly R. Munkittrick

**Affiliations:** ^1^ Water Science and Technology Directorate, Environment and Climate Change Canada, Burlington, ON, Canada; ^2^ Department of Biology, University of Waterloo, Waterloo, ON, Canada; ^3^ Great Lakes Toxicology and Ecology Division, Center for Computational Toxicology and Exposure, US Environmental Protection Agency, Ann Arbor, MI, United States; ^4^ Biological Sciences, University of Calgary, Calgary, AB, Canada

**Keywords:** recovery, wild fish health, reproductive alterations, ecosystem recovery, process change

## Abstract

Jackfish Bay is an isolated bay on the north shore of Lake Superior, Canada that has received effluent from a large bleached-kraft pulp mill since the 1940s. Studies conducted in the late 1980s found evidence of reductions in sex steroid hormone levels in multiple fish species living in the Bay, and increased growth, condition and relative liver weights, with a reduction in internal fat storage, reduced gonadal sizes, delayed sexual maturation, and altered levels of circulating sex steroid hormones in white sucker (*Catostomus commersonii*). These early studies provided some of the first pieces of evidence of endocrine disruption in wild animals. Studies on white sucker have continued at Jackfish Bay, monitoring fish health after the installation of secondary waste treatment (1989), changes in the pulp bleaching process (1990s), during facility maintenance shutdowns and during a series of facility closures associated with changing ownership (2000s), and were carried through to 2019 resulting in a 30-year study of fish health impacts, endocrine disruption, chemical exposure, and ecosystem recovery. The objective of the present study was to summarize and understand more than 75 physiological, endocrine, chemical and whole organism endpoints that have been studied providing important context for the complexity of endocrine responses, species differences, and challenges with extrapolation. Differences in body size, liver size, gonad size and condition persist, although changes in liver and gonad indices are much smaller than in the early years. Population modeling of the initial reproductive alterations predicted a 30% reduction in the population size, however with improvements over the last couple of decades those population impacts improved considerably. Reflection on these 30 years of detailed studies, on environmental conditions, physiological, and whole organism endpoints, gives insight into the complexity of endocrine responses to environmental change and mitigation.

## Introduction

Jackfish Bay is an isolated bay on the north shore of Lake Superior, Canada, and it has received the effluent from a large bleached-kraft pulp mill at Terrace Bay, Ontario since the 1940s. In the late 1980s studies on the effects of pulp mill effluent entering Lake Superior at Jackfish Bay documented delayed sexual maturity, reductions in gonad size, sex steroid hormones and secondary sex characteristics in white sucker (*Catostomus commersonii*; 1, 2). Studies also confirmed a delay in sexual maturity in lake whitefish (*Coregonus clupeaformis*) at Jackfish Bay (3), and reproductive impacts in white sucker at a number of other mills (4). Intensive study at Jackfish Bay documented disruptions at multiple sites along the pituitary-gonad axis including reductions in circulating levels of pituitary gonadotropins, responsiveness to GnRH (5), reductions in steroid hormone production (6), and several disruptions in the steroid hormone synthetic pathway (7). Modeling of the reproductive changes documented during the late 1980s and early 1990s at Jackfish Bay suggested a 34% to 51% annual decrease in recruitment of white sucker (8).

These studies confirmed earlier studies in Sweden which suggested that pulp mill effluents releasing organically bound chlorine and its chemical constituents could cause impacts on the growth and reproduction of fish at much lower environmental concentrations than previously thought (9). Broader Canadian studies showed that impacts were not present all pulp mill effluent-exposed sites and impacts appeared to be site-specific and affected by a number of potential modifying factors including differences in receiving environment affecting exposure concentrations and undefined differences between mills in processes and effluent treatment processes (4, 10, 11). Although there have been changes in fish in response to mitigation, linking specific chemicals in pulp mill effluents to effects has been challenging and elusive (12). Studies at Jackfish Bay, Ontario, Canada, and those done at Norrsundet in Sweden, increased global awareness of the potential for industrial effluents to impact reproductive processes in fish, and prompted studies in more than 15 countries (11).

Due to concerns about the potential for impacts downstream of pulp mills, Canada developed requirements for a regulated, cyclical, Environmental Effects Monitoring (EEM) program (11). The Canadian EEM program involves rolling cycles of study design, monitoring, and reporting to ensure the effectiveness of regulatory protection. The EEM program introduced across Canada showed that the most consistent pattern seen in Cycle 2 (1996–2000) and Cycle 3 (2000–2003) was increased condition factor and liver size and decreased gonad size relative to reference sites; more than 60 species of fish have been used in the EEM program (13–15). This was the same pattern that was initially reported in white sucker collected from Jackfish Bay during the early fall collections in the 1980s and early 1990s (prior to treatment upgrades). Studies have continued at Jackfish Bay during a series of waste treatment and process improvements.

The updated Canadian regulations of the early 1990s also prompted a series of process changes and modernizations in the Canadian pulp and paper industry, which spent over $8 billion in improvements to reduce contaminants (16, 17). Pulping process changes were also undertaken to reduce the potential for the formation of polychlorinated dibenzo-*p*-dioxins and dibenzofurans (PCDD/F), including the substitution of chlorine dioxide (ClO_2_) or other bleaching agents for elemental chlorine (18). Numerous studies have attempted to follow fish reproduction responses to these numerous improvements and process changes (10, 19–24). Unfortunately, the establishment of linkages between production changes and improvements on fish reproductive performance has been difficult to establish. Many mills performed multiple process changes simultaneously, therefore making it difficult to attribute specific changes in the mill to changes in fish responses in the receiving environment (25). Despite many improvements, subtle effects on fish reproduction have persisted (11, 19, 24). When effects were detected and confirmed above defined critical effect sizes, the EEM program required the initiation of Investigation of Cause (IOC) and Investigation of Solution (IOS) studies. Several mills demonstrating metabolic disruption (i.e. energy put into storage instead of reproductive development), participated in a joint government, industry and academia IOC study. A short-term fathead minnow (*Pimephales promelas*) reproductive test (26) was applied to test effluents from across the country (27, 28). Kraft and mechanical pulp mill effluents containing less than 20 mg/L BOD were found to have the greatest probability of having no effects on egg production. Results from laboratory studies demonstrated the importance of both in-plant measures for controlling the loss of organics as well as the optimum operation of biological effluent treatment for eliminating effluent-related effects on fish reproduction (egg production) in the laboratory (27). Best management practices were developed and provided to industry, and field studies within the EEM program continue to evaluate potential success of these practices.

Current economic conditions, coupled with the expansion of lower cost pulp producers in South America, have resulted in large scale changes in the Canadian pulp and paper industry including outsourcing, closures, reductions in costs, and merging of companies (29, 30). These changes have resulted in closure of a number of mills where significant past studies had taken place, and created the opportunity to follow changes in fish responses resulting from closures at Smooth Rock Falls, Ontario (21, 31), and Miramichi, New Brunswick (32–34). The economic conditions inevitably also affected the Terrace Bay pulp mill, resulting in several mill closures, although this mill has continued to operate and discharge effluent into Jackfish Bay.

Fish responses at Jackfish Bay were followed in association with installation of secondary waste treatment (1989), and changes in the pulp bleaching process (1990s), as well during facility maintenance shutdowns and a series of facility closures associated with changing ownership (2000s). Collections at Jackfish Bay in 2018 and 2019 extend the time series of spring and fall fish collections allowing for the examination of potential recovery, resulting from more recent changes in effluent quality. This now represents a unique 30+ year study of fish health impacts, endocrine disruption, chemical exposure, and ecosystem recovery. Although the earlier studies at Jackfish Bay (1988–2007) were summarized by Bowron et al. (35) much of the data since 2007 has not been previously published. The present study therefore synthesizes data from a wide variety of past and recent studies (**Table 1**) into a chronological summary of the responses of fish populations to numerous changes over three decades and provides important perspectives and insights into understanding long-term variability, the ecological relevance of changes, reference site variability and normal ranges that affect the design and interpretation of monitoring studies. The objective of the present study was to summarize and understand the, more than 75 physiological, endocrine, chemical and whole organism endpoints that have been studied providing important context for the complexity of endocrine responses, species differences, and challenges with extrapolation.

**Table 1:** Summary of main endpoints from studies that have been conducted at Jackfish Bay.

Season	Year	Endpoints	Author
Spring	1988	Population level characteristics, cyp450 induction, and sex steroid levels	Munkittrick et al. (2)
	1989	Population level characteristics, cyp450 induction, and sex steroid levels	McMaster et al. (1)
	1990	Pituitary-gonadal axis disruption	Van Der Kraak et al. (5)
	1991	Acute stress response Steroid biosynthetic pathway	McMaster et al. (36) McMaster et al. (6)
	1992	Stress response	Jardine et al. (37)
	1993	Stress response Assessing if fish exposed to various organic compounds show similar reproductive alterations 2,3,7,8-TCDD toxic equivalent concentrations MFO induction	Jardine et al. (37) McMaster et al. (38) Van den Heuvel et al. (39) Parrott et al. (40)
	1994	Assessing if fish exposed to various organic compounds show similar reproductive alterations	McMaster et al. (38)
	1996	Heat shock protein-70 expression and ovarian follicular apoptosis	Janz et al. (41)
	1996, 1999, 2001	Stress response and free radical production	Oakes et al. (42)
	2008, 2011, 2013, 2018, 2019	Population level characteristics, cyp450 induction, and sex steroid levels	Ussery et al., (this manuscript)
			
Fall	1988	Population level characteristics, cyp450 induction, and sex steroid levels	Munkittrick et al. (2)
	1989	Population level characteristics, cyp450 induction, and sex steroid levels	McMaster et al. (1)
	1990	Response of cyp450 induction, gonadal development, and sex steroids in white sucker and longnose sucker (*Catostomus catostomus*) Response of cyp450 induction, gonadal development, and sex steroids in lake whitefish (*Coregonus clupeaformis*)	Munkittrick et al. (19) Munkittrick et al. (3)
	1991	Steroid biosynthetic pathway Cyp450 induction Assessing if fish exposed to various organic compounds show similar reproductive alterations	McMaster et al. (6) Munkittrick et al. (43) McMaster et al. (38)
	1992	Assessing if fish exposed to various organic compounds show similar reproductive alterations 2,3,7,8-TCDD toxic equivalent concentrations	McMaster et al. (38) Van den Heuvel et al. (39)
	1993	Assessing if fish exposed to various organic compounds show similar reproductive alterations	McMaster et al. (38)
	1995	EEM Cycle 1	BEAK (44)
	1999	EEM Cycle 2	BEAK (45)
	2000	Stress response and free radical production	Oakes et al., 2 (42)
	2002	EEM Cycle 3	Stantec (46)
	2004	EEM Cycle 4	Ecometrix (47)
	2008, 2011, 2013, 2018, 2019	Population level characteristics, cyp450 induction, and sex steroid levels	Ussery et al., (this manuscript)
	2018	Histological impacts of white sucker at Jackfish Bay	Williams et al. (48),

## Materials And Methods

Long-term fish health studies at Jackfish Bay Lake Superior from 1988 – 2007 summarized in Bowron et al. (35) demonstrated some recovery in fish health endpoints following both process and treatment changes as well as during a mill shutdown period. Over the next 12 years, a second longer shutdown, closure during an ownership change, and additional process improvements has occurred. The Jackfish Bay Area of Concern (AOC) was also re-designated as an Area in Recovery by the International Joint Commission in 2011. Six additional spring and fall collections of white sucker health data have been completed since the Bowron et al. (35) review. With the exception of PCDD/F levels from fish collected in 2011 and 2012 (49), data collected since 2008 have not been previously published. The fish collection and analysis methods described by Bowron et al. (35) have remained relatively constant over the time period of the study (1988–2019) and additional specific and detailed methodologies for biochemical endpoints can be found in earlier studies as summarized in **Table 1**.

### Study Site

**Figure 1:**
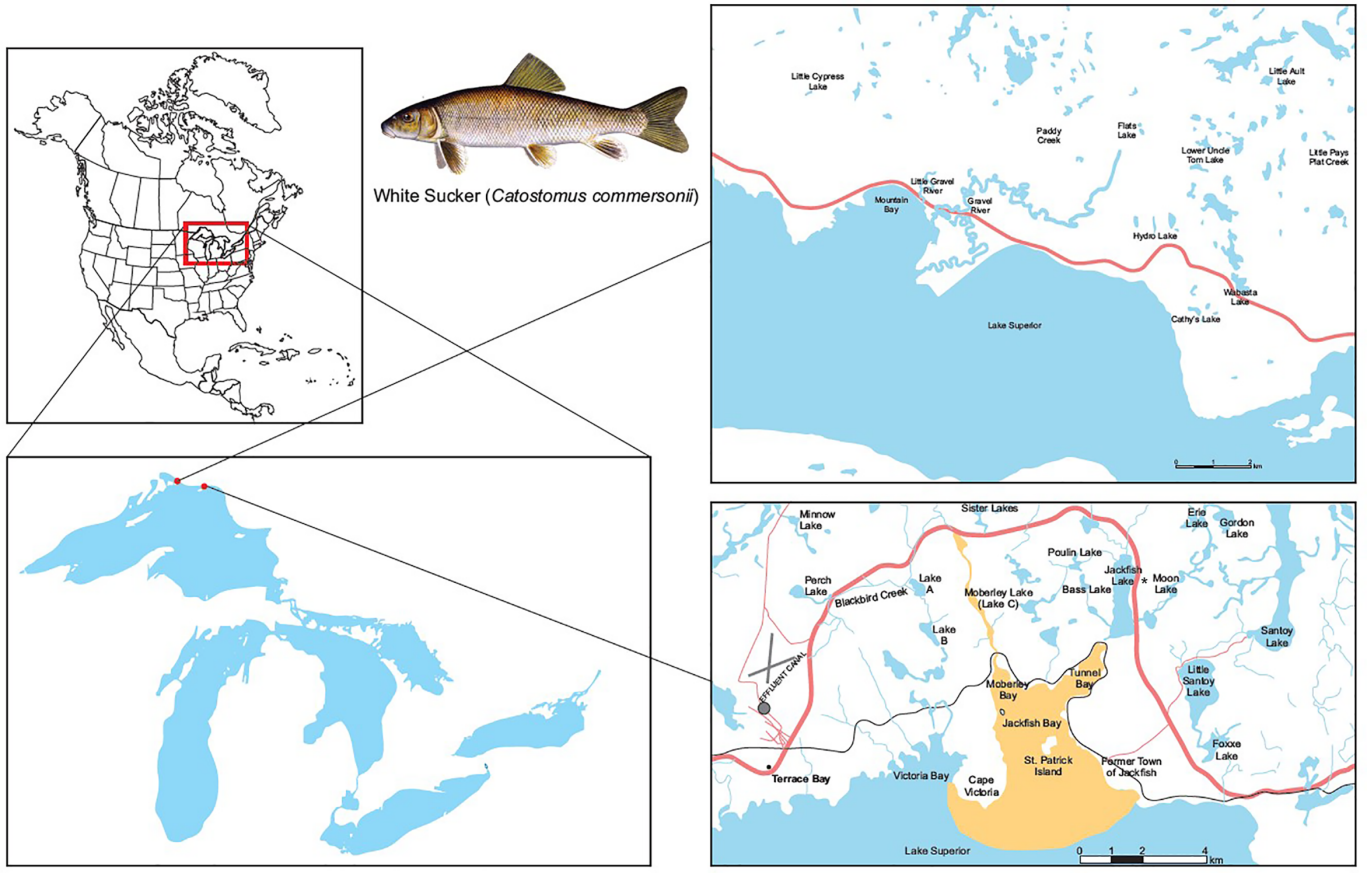
Map of study site at Jackfish Bay on the north shore of Lake Superior, Canada. The Jackfish Bay area of concern in recovery includes Blackbird Creek, Moberley Bay, Tunnel Bay, and Jackfish Bay proper. Effluent enters Blackbird Creek and flows into Lake Superior at Jackfish Bay (48°50′N; 86°58′W). Mountain Bay (46°56′N; 87°58′W) located 60 km away from Jackfish Bay was used as the reference site for the 30 year monitoring study. The * identifies the spawning site for Jackfish Bay white sucker at Sawmill Creek which is in the flow between Moon Lake and Jackfish Lake.

The pulp mill located at Terrace Bay, Ontario began operation in 1948 producing 320 air dried metric tonne (ADMT) of softwood pulp per day (**Table 2**). Currently AV Terrace Bay is a northern bleached softwood kraft mill producing 1,154 ADMT. The mill discharges effluent into Blackbird Creek, which carries the effluent 15 km to Jackfish Bay on Lake Superior (**Figure 1**). In 2019, the average monthly discharge into the creek was approximately 91,000 m^3^/d. In the early 1900s, the town of Jackfish was an important commercial fishing location and an essential railway stop for coal refueling. In the 1940s diesel train engines came into use and the town of Jackfish became a ghost town and there are now no permanent residents or other discharges into Jackfish Bay. While early studies included additional pulp mills and reference sites (4, 50), most of the focus of the 30-year study reported here is on Jackfish Bay, with a similar bay to the west in Lake Superior, Mountain Bay, used as a reference site.

**Table 2:** Summary of major process changes with effluent quality at Jackfish Bay. Information in table adapted from Bowron et al. (35).

Year	Process Change
1948	Mill constructed, operations commenced: 320 air-dried metric ton/d (ADMT/d) hardwood.
1958	Chlorine dioxide was added to mill bleaching circuit.
1959	Cold bleaching process was added.
1972	Mill expanded, incorporating two lines of hot bleaching. Increased capacity to 400 ADMT/d. (1972–78 mercury anode utilized in the formation of Cl_2_ gas for bleaching).
1973	Recovery boiler installed.
1975	There are 3 small lakes before Lake Superior (A,B,C); Blackbird Creek diverted around Lake A. Increased storage to 435 ADMT/d.
1978	Addition of new mill/plant and bleaching. Capacity increased to 1135 ADMT/d. Two reactor clarifiers for alkaline sewage were added as part of newly installed primary treatment plant. Dry debarking added to Mill No. 2.
1979	Addition of clarifier for alkaline sewage.
1981	Major mill reconstruction after accidental fire: addition of condensate stripper turpentine decanter, NCG collection and destruction system, by-pass of domestic sewage treatment plant and clarifier screening system installed.
1982	Cooling water recycle system for kiln/causticizing area installed.
1984	Spill control completed in Mill No. 2, improved soap recovery, increased chlorine dioxide substitution, and additional clarifier and improvements to Mill No. 2 brownstock washing. Mill No. 1 dedicated to hardwood, polymer feed system for causticizer.
1985	Mill No. 2 brownstock closure; spill control system for Mill No. 1 installed; E_0_ stage added to Mill No. 2 bleachery, new instrumentation for bleachery to decrease chemical use.
1986	Modification of Mill No. 1 brownstock washers completed: which improved soap recovery, foam control and vacuum improvements.
1989	Secondary treatment plant installed.
1990	Increased chlorine dioxide substitution; hypochlorite replaced with Papricycle.
1991	Chlorine strength analyzers added; addition of recirculation piping, new chip screening plant and hot water stave replaced; increased production by 45 ADMT/d (now ~ 1180 ADMT/d).
1993	Concentrator addition for Mill No. 2 recovery boiler (black liquor): steam operated, two effect concentrators in increase liquor solids from 63% to 75% and eliminates the cascade evaporator; low liquor concentration and moist, lower temperature combustion air from cascade evaporator leads to formation of total reduced sulfur compounds; resulted in improved air quality.
1994	Replacement of 250 m section wooden stave piping.
1995	Cl generator was updated from R3 to R8 process continual production of ECF pulp (mill gradual increase to utilization of 100% ECF) in both the hardwood (No. 1) and softwood (No. 2) mills.
1996	Additional mature wood purchased: which lessened lignin content and subsequent sulfur lignin by-products.
1997	Hydrogen peroxide started in Mill No. 2 in second E_0_ stage.
1998	Hydrogen peroxide started in Mill No. 2 in first E_0_ stage; Mill No. 1 switched to batch softwood production for short runs; Mill No. 1 switched over to 100% ECF bleaching in October.
1999	Mill No. 2 switched to 100% ECF bleaching in April.
2000	New brownstock washing showers added in September.
2003	Reduced absorbable organic halides (AOX) by 47% by modifying third stage of bleaching. Acid activated oxygen bleaching stage with 2 reactors was installed in third stage of Mill No. 2.
2005	Mill No. 1 shutdown April 1.
2006	Mill No. 2 shutdown February 20 and restarted September 19.
2007	Mill No. 1 restarted on softwood. Request by Ontario Ministry of Environment to Terrace Bay Pulp Inc. (TBPI) to approve their bark resource pile as waste transfer/processing site or provide information to allow for the closure of the area as a waste site by July 31. TBPI was required to install a groundwater monitoring well and to conduct ground water and surface water monitoring to provide report to the Ministry by 2008.
2008	Addition of new steam turbine: allowing 1 month shutdown from November to December.
2009	Mill No. 1 shutdown; Mill No. 2 reduced production, then completely shut down in February.
2010	Mill reopened in October.
2011	Mill temporarily shut down in October.
2011	As of May 2011, Jackfish Bay has been designated an Area of Concern in Recovery (AOCiR) under the Canada–US Great Lakes Water Quality Agreement.
2012	Mill switched management: bought out by Aditya Birla Group in July 2012. Operating under Canadian division of AV Terrace Bay. Northern bleached softwood began in October 2012. Plans to switch process type away from bleached kraft in next few years to dissolved pulp production (used in manufacture of rayon).
2013 to present	Operating as northern bleached softwood kraft.

In 1987 Jackfish Bay was listed as a Great Lakes AOC under the Canada-United States Great Lakes Water Quality Agreement because of the impairment of beneficial uses associated with the effluent discharged from the bleached kraft pulp mill located in Terrace Bay (49, 51). The AOC included the embayments of Jackfish Bay, Moberly Bay and Tunnel Bay, and a 14-km stretch of Blackbird Creek that receives effluent from the mill (51; **Figure 1**).

### Fish Sampling

Reproductive studies on white sucker began in 1988 at Jackfish Bay, continuing intermittently until 2007 during both prespawning (spring) and gonadal recrudescence (fall) periods (35). Fall sampling timing varied slightly between the years, as the recrudescence collection period was moved during early years of the studies from early August to early September to allow for further gonadal development to occur. Studies continued intermittently during both spring and fall after 2007 with the timing of recent fall studies conducted at the end of August or early September.

Prespawning samples were collected in May by setting hoop nets across spawning streams and collecting fish as they ascended to the spawning beds at both Sawmill Creek (exposure site) and Little Gravel River (reference site) (**Figure 1**); the timing of the spawning runs varied between the first and last weeks of May. During the first year, 100 males and 100 females were collected from each site to examine age to maturation, and later studies targeted 20 males and 20 females, with the exception of 1996 (100 males and 100 females), 2006 (50, 50) and 2011 (100, 100) (see **Tables 3** **,** **4** for actual sample sizes for each year). Fish were removed from hoop nets and held in cages until sampling that same day.

**Table 3:** Age, length, weight, condition factor (K), gonadosomatic index (GSI), liver somatic index (LSI), and fecundity (total number of eggs) of female white sucker (*Catostomus commersoni*) from Jackfish Bay and Mountain Bay, collected during pre-spawning (spring) from 1988 to 2019.

Site	Date	Age (year)	Length (cm)	Weight (g)	GSI^a^	LSI^b^	K^c^	Fecundity
JFB	1988	10.1 ± 0.4 (100)*	39.8 ± 0.3 (100)*	964.1 ± 22.3 (100)*	10.9 ± 0.5 (34)*		1.50 ± 0.01 (100)*	17136 ± 1315 (27)*
	1989	10.7 ± 0.5 (45)*	40.7 ± 0.4 (45)	993.4 ± 40.9 (25)	10.6 ± 0.5 (24)*	2.23 ± 0.07 (14)*	1.49 ± 0.02 (25)*	24397 ± 1440 (23)
	1990	9.9 ± 0.5 (32)*	40.4 ± 0.5 (33)*	956.0 ± 34.4 (33)	11.5 ± 0.3 (32)*	1.76 ± 0.05 (23)	1.43 ± 0.02 (33)*	22909 ± 1130 (28)
	1991	11.4 ± 0.3 (83)*	40.3 ± 0.3 (83)*	962.2 + 22.8 (83)	11.5 ± 0.3 (83)*	2.13 ± 0.06 (82)*	1.39 ± 0.02 (83)*	19019 ± 818 (27)
	1992	12.4 ± 0.3 (60)*	41.0 ± 0.3 (63)*	954.9 ± 25.4 (49)*	11.1 ± 0.3 (48)*	1.82 ± 0.07 (37)*	1.39 ± 0.02 (49)*	
	1993		40.3 ± 0.3 (85)*	970.0 ± 26.1 (85)	12.0 ± 0.3 (78)^d^	1.71 ± 0.04 (80)*	1.2646 ± 0.01 (85)*	
	1996	13.4 ± 0.3 (101)	42.1 ± 0.3 (101)*	1091.4 ± 22.9 (101)*	12.9 ± 0.2 (100)*	1.96 ± 0.03 (100)*	1.44 ± 0.01 (101)*	27487 ± 1152 (54)
	1999		42.7 ± 0.7 (20)*	1237.6 ± 67.0 (20)	14.2 ± 0.5 (20)	1.52 ± 0.10 (20)	1.56 ± 0.02 (20)*	22550 ± 2843 (20)*
	2001	9.6 ± 0.7 (20)	41.6 ± 0.7 (20)	1072.1 ± 51.1 (20)	12.0 ± 0.4 (20)	1.79 ± 0.05 (20)	1.47 ± 0.01 (20)*	24574 ± 1450 (20)
	2003	8.9 ± 1.0 (19)*	42.4 ± 0.7 (20)*	1156.2 ± 60.3 (20)*	12.5 ± 0.3 (20)*	1.80 ± 0.06 (20)*	1.50 ± 0.03 (20)*	29249 ± 1872 (20)
	2006	10.2 ± 0.4 (50)*	41.3 ± 0.5 (50)*	1039.7 ± 39.8 (50)*	12.6 ± 0.3 (49)	1.56 ± 0.03 (50)^d^	1.44 ± 0.01 (50)*	27422 ± 1946 (20)
	2007	10.7 ± 0.6 (30)*	43.6 ± 0.7 (30)	1223.3 ± 45.1 (30)	13.7 ± 0.3 (29)^d^	1.86 ± 0.05 (30)	1.47 ± 0.03 (30)*	32398 ± 2182 (20)*
	2008	7.3 ± 0.5 (21)*	41.9 ± 0.6 (22)*	1105.1 ± 50.6 (22)*	13.0 ± 0.3 (22)*	1.30 ± 0.02 (22)	1.49 ± 0.02 (22)	27455 ± 1748 (22)
	2011	10.2 ± 0.2 (103)*	43.8 ± 0.3 (103)	1355.9 ± 25.1 (103)*	14.5 ± 0.2 (103)*	1.93 ± 0.02 (103)^d^	1.59 ± 0.02 (103)^d^	29589 ± 812 (99)*
	2012	11.0 ± 0.4 (20)*	44.1 ± 0.5 (20)	1339.2 ± 44.1 (20)	13.6 ± 0.3 (20)^d^	1.87 ± 0.04 (20)*	1.56 ± 0.02 (20)*	36262 ± 1994 (18)*
	2013	9.1 ± 0.5 (33)*	43.5 ± 0.6 (33)*	1275.4 ± 58.0 (33)*	13.8 ± 0.4 (33)*	1.83 ± 0.05 (33)	1.51 ± 0.02 (33)^d^	33879 ± 1769 (33)*
	2018	7.8 ± 0.4 (20)	45.3 ± 0.8 (20)*	1421.2 ± 76.1 (20)*	13.3 ± 0.3 (20)	1.87 ± 0.06 (20)	1.50 ± 0.02 (20)*	37191 ± 2392 (20)*
	2019	8.1 ± 0.3 (20)	42.6 ± 0.7 (20)	1060.6 ± 57.2 (20)	13.7 ± 0.6 (20)	2.03 ± 0.08 (20)*	1.35 ± 0.03 (20)	32720 ± 2105 (19)*
MTB	1988	8.4 ± 0.4 (101)	41.9 ± 0.3 (101)	1067.7 ± 20.4 (101)	13.8 ± 0.3 (45)		1.44 ± 0.01 (101)	20556 ± 800 (37)
	1989	8.4 ± 0.6 (46)	41.9 ± 0.5 (46)	973.2 ± 39.9 (26)	13.1 ± 0.4 (26)	2.42 ± 0.07 (15)	1.39 ± 0.01 (26)	24388 ± 1163 (26)
	1990	7.9 ± 0.3 (29)	42.6 ± 0.5 (31)	1039.9 ± 29.2 (31)	13.5 ± 0.4 (31)	1.62 ± 0.05 (30)	1.34 ± 0.01 (31)	21386 ± 1082 (22)
	1991	8.6 ± 0.1 (88)	41.5 ± 0.2 (88)	924.9 ± 13.6 (88)	12.9 ± 0.2 (86)	1.78 ± 0.03 (88)	1.29 ± 0.01 (88)	17856 ± 772 (33)
	1992	8.9 ± 0.4 (19)	42.2 ± 0.3 (53)	1019.4 ± 19.7 (53)	13.5 ± 0.3 (53)	1.95 ± 0.04 (40)	1.35 ± 0.01 (53)	
	1993		41.9 ± 0.3 (76)	987.7 ± 17.9 (76)	12.4 ± 0.6 (34)	1.55 ± 0.03 (71)	1.33 ± 0.01 (76)	
	1996	11.9 ± 0.3 (103)	43.7 ± 0.2 (104)	1172.5 ± 19.4 (104)	14.4 ± 0.2 (104)	2.15 ± 0.03 (102)	1.39 ± 0.01 (104)	28262 ± 923 (51)
	1999		44.6 ± 0.6 (22)	1268.9 ± 58.6 (21)	15.5 ± 0.6 (21)	1.79 ± 0.06 (21)	1.40 ± 0.02 (21)	14348 ± 1150 (20)
	2001	8.9 ± 0.9 (16)	43.6 ± 0.8 (20)	1156.8 ± 71.6 (20)	13.3 ± 0.5 (20)	2.24 ± 0.05 (20)	1.36 ± 0.02 (20)	24693 ± 1613 (20)
	2003	13.6 ± 1.1 (19)	47.0 ± 0.7 (20)	1417.7 ± 51.9 (20)	14.5 ± 0.4 (20)	1.89 ± 0.05 (20)	1.36 ± 0.03 (20)	28975 ± 1304 (20)
	2006	13.8 ± 0.8 (37)	45.0 ± 0.5 (37)	1245.7 ± 40.6 (37)	14.5 ± 0.5 (37)	1.68 ± 0.06 (37)	1.35 ± 0.02 (37)	29525 ± 1577 (20)
	2007	13.7 ± 1.0 (20)	44.7 ± 0.7 (20)	1212.2 ± 54.3 (20)	14.8 ± 0.3 (20)	1.83 ± 0.06 (20)	1.34 ± 0.02 (20)	26192 ± 860 (20)
	2008	13.2 ± 1.1 (20)	45.4 ± 0.4 (20)	1359.3 ± 42.1 (20)	15.5 ± 0.4 (20)	1.99 ± 0.07 (20)	1.44 ± 0.02 (20)	28932 ± 1217 (20)
	2011	11.7 ± 0.4 (101)	43.9 ± 0.3 (101)	1228.7 ± 28.5 (101)	15.0 ± 0.3 (101)	1.92 ± 0.03 (101)	1.42 ± 0.01 (101)	22250 ± 752 (99)
	2012	13.4 ± 1.1 (19)	45.1 ± 0.7 (20)	1281.7 ± 56.2 (20)	15.4 ± 0.5 (22)	1.63 ± 0.06 (20)	1.38 ± 0.02 (20)	25547 ± 1981 (16)
	2013	7.2 ± 0.5 (20)	41.0 ± 0.6 (20)	950.7 ± 47.8 (20)	12.5 ± 0.4 (20)	2.23 ± 0.02 (19)	1.36 ± 0.02 (20)	20290 ± 1333 (19)
	2018	6.8 ± 0.2 (20)	41.9 ± 0.5 (20)	993.6 ± 38.2 (20)	14.1 ± 0.3 (20)	1.92 ± 0.06 (20)	1.35 ± 0.02 (20)	22839 ± 921 (20)
	2019	8.2 ± 0.3 (20)	42.8 ± 0.6 (20)	1018.5 ± 42.7 (20)	13.2 ± 0.6 (20)	1.67 ± 0.06 (20)	1.29 ± 0.02 (20)	21235 ± 1309 (20)

Values are reported as mean ± SE (n). Shaded cells are either below (light grey) or above (dark grey) normal range for Mountain Bay.

*Significant difference between sites (p ≤ 0.05) within years; ^a^ GSI = 100* (gonad weight/body weight); ^b^ LSI = 100* (liver weight/body weight); ^c^ K = 100*(weight/length3); ^d^ Represents an interaction between sites; All data before 2008 published in Bowron et al. (35).

**Table 4:** Age, length, weight, condition factor (K), gonadosomatic index (GSI), and liver somatic index (LSI) of male white sucker (*Catostomus commersoni*) from Jackfish Bay (JFB) and Mountain Bay (MTB), collected during pre-spawning (spring) from 1988 to 2019.

Site	Date	Age (year)	Length (cm)	Weight (g)	GSI^a^	LSI^b^	K^c^
JFB	1988	9.0 ± 0.3 (100)*	36.9 ± 0.3 (100)*	741.4 ± 18.4 (100)			1.44 ±0.01 (100)*
	1989	8.4 ± 0.4 (38)*	36.7 ± 0.4 (38)*	697.5 ± 34.4 (15)	4.22 ± 0.23 (15)	2.04 ± 0.06 (15)	1.46 ± 0.02 (15)*
	1990	8.7 ± 0.4 (23)	37.6 ± 0.5 (23)*	705.1 ± 29.1 (23)*	4.44 ± 0.21 (23)	1.55 ± 0.13 (23)	1.31 ± 0.02 (23)*
	1991	9.7 ± 0.4 (18)	37.1 ± 0.6 (18)	711.1 ± 33.7 (18)	4.13 ± 0.21 (18)*	1.56 ± 0.06 (18)*	1.38 ± 0.01 (18)*
	1992	10.5 ± 0.3 (51)	37.2 ± 0.4 (52)*	673.2 ± 24.8 (41)*	3.89 ± 0.16 (41)*	1.45 ± 0.11 (31)	1.29 ± 0.02 (41)*
	1993		36.8 ± 0.3 (85)*	699.8 ± 17.3 (85)*	3.94 ± 0.11 (80)*	1.48 ± 0.03 (80)*	1.39 ± 0.01 (85)*
	1996	10.1 ± 0.3 (104)	37.7 ± 0.3 (106)*	728.1 ± 17.8 (106)*	4.55 ± 0.09 (105)*	1.38 ± 0.04 (106)*	1.33 ± 0.01 (106)*
	1999		37.8 ± 0.7 (24)	752.1 ± 48.7 (24)	4.81 ± 0.40 (24)	1.15 ± 0.06 (24)*	1.35 ± 0.04 (24)*
	2001	7.9 ± 0.7 (20)*	37.8 ± 0.7 (20)*	788.4 ± 45.1 (20)	4.27 ± 0.23 (20)	1.42 ± 0.07 (20)	1.43 ± 0.03 (20)*
	2003	8.6 ± 1.0 (18)*	38.8 ± 0.4 (20)*	826.5 ± 24.5 (20)*	4.38 ± 0.17 (20)	1.23 ± 0.05 (20)	1.41 ± 0.01 (20)*
	2006	9.9 ± 0.5 (50)	37.9 ± 0.5 (50)*	784.2 ± 35.2 (50)	4.71 ± 0.55 (50)	1.31 ± 0.03 (49)*	1.40 ± 0.01 (50)*
	2007	10.6 ± 0.5 (20)	40.4 ± 0.5 (20)	968.0 ± 39.2 (20)*	4.79 ± 0.16 (20)	1.35 ± 0.06 (20)*	1.45 ± 0.02 (20)*
	2008	6.3 ± 0.4 (21)*	38.6 ± 0.4 (21)*	825.3 ± 26.2 (21)*	4.02 ± 0.17 (21)^d^	1.53 ± 0.05 (20)^d^	1.43 ± 0.03 (21)
	2011	8.0 ± 0.3 (98)	37.6 ± 0.4 (98)	806.1 ± 29.0 (98)	4.46 ± 0.11 (98)^d^	1.43 ± 0.03 (98)^d^	1.44 ± 0.01 (98)*
	2012	9.2 ± 0.5 (20)	39.4 ± 0.5 (20)	888.9 ± 32.2 (20)	4.89 ± 0.20 (20)	1.43 ± 0.06 (20)*	1.44 ± 0.02 (20)*
	2013	7.6 ± 0.5 (32)	39.0 ± 0.6 (32)	848.6 ± 39.1 (32)*	5.00 ± 0.13 (32)	1.52 ± 0.04 (32)^d^	1.40 ± 0.01 (32)^d^
	2018	6.3 ± 0.2 (20)	38.2 ± 0.8 (20)	793.6 ± 51.5 (20)	4.06 ± 0.15 (20)*	1.37 ± 0.05 (20)	1.40 ± 0.02 (20)*
	2019	7.6 ± 0.2 (20)	39.1 ± 0.6 (20)	786.9 ± 37.8 (20)*	4.66 ± 0.28 (20)^d^	1.49 ± 0.09 (20)	1.30 ± 0.02 (20)
MTB	1988	7.7 ± 0.4 (99)	38.2 ± 0.3 (99)	786.4 ± 17.3 (99)			1.39 ± 0.01 (99)
	1989	7.2 ± 0.4 (45)	38.3 ± 0.5 (45)	780.5 ± 38.4 (15)	4.84 ± 0.25 (14)	2.29 ± 0.25 (14)	1.30 ± 0.02 (15)
	1990	8.3 ± 0.4 (31)	40.1 ± 0.4 (32)	831.6 ± 26.9 (32)	4.94 ± 0.14 (31)	1.61 ± 0.10 (32)	1.28 ± 0.02 (32)
	1991	8.6 ± 0.8 (17)	37.9 ± 0.3 (17)	707.5 ± 17.5 (17)	4.97 ± 0.14 (17)	1.61 ± 0.06 (17)	1.29 ± 0.02 (17)
	1992	9.4 ± 0.7 (18)	39.0 ± 0.3 (48)	789.0 ± 15.8 (48)	4.75 ± 0.11 (48)	1.69 ± 0.04 (36)	1.32 ± 0.01 (48)
	1993		38.9 ± 0.4 (42)	766.3 ± 21.2 (42)	4.47 ± 0.15 (36)	1.64 ± 0.06 (37)	1.28 ± 0.01 (42)
	1996	12.7 ± 0.3 (102)	41.0 ± 0.2 (102)	922.4 ± 14.2 (102)	5.64 ± 0.08 (102)	1.80 ± 0.03 (102)	1.33 ± 0.01 (102)
	1999		39.4 ± 0.7 (20)	816.9 ± 44.6 (20)	5.50 ± 0.22 (20)	1.71 ± 0.08 (20)	1.31 ± 0.01 (20)
	2001	11.9 ± 1.4 (8)	40.3 ± 0.5 (20)	854.9 ± 34.9 (20)	4.68 ± 0.19 (20)	1.57 ± 0.06 (20)	1.29 ± 0.02 (20)
	2003	13.6 ± 1.0 (20)	43.2 ± 0.5 (20)	1030.8 ± 31.9 (20)	5.30 ± 0.20 (20)	1.60 ± 0.07 (20)	1.28 ± 0.02 (20)
	2006	11.0 ± 0.8 (31)	40.0 ± 0.4 (31)	827.5 ± 25.5 (31)	4.83 ± 0.23 (31)	1.61 ± 0.07 (31)	1.28 ± 0.01 (27)
	2007	11.7 ± 1.2 (20)	39.7 ± 0.6 (20)	778.6 ± 32.9 (20)	5.31 ± 0.20 (20)	1.63 ± 0.07 (20)	1.24 ± 0.02 (20)
	2008	12.3 ± 0.9 (20)	42.0 ± 0.3 (20)	981.7 ± 27.5 (20)	4.88 ± 0.21 (20)	1.70 ± 0.05 (20)	1.32 ± 0.02 (20)
	2011	9.0 ± 0.4 (98)	38.3 ± 0.3 (98)	761.3 ± 20.1 (98)	4.90 ± 0.18 (98)	1.94 ± 0.06 (98)	1.33 ± 0.01 (98)
	2012	9.5 ± 0.9 (20)	39.6 ± 0.7 (20)	824.1 ± 42.2 (20)	4.92 ± 0.17 (20)	1.69 ± 0.06 (20)	1.31 ± 0.02 (20)
	2013	6.3 ± 0.3 (19)	37.5 ± 0.5 (19)	677.4 ± 27.4 (19)	4.55 ± 0.13 (19)	1.81 ± 0.07 (19)	1.28 ± 0.04 (19)
	2018	6.1 ± 0.2 (20)	38.0 ± 0.4 (20)	673.4 ± 21.7 (20)	4.76 ± 0.12 (20)	1.49 ± 0.06 (20)	1.22 ± 0.01 (20)
	2019	7.5 ± 0.2 (22)	37.9 ± 0.5 (22)	690.0 ± 31.2 (22)	4.74 ± 0.20 (22)	1.47 ± 0.07 (21)	1.25 ± 0.02 (22)

Values are reported as mean ± SE (n). Shaded cells are either below (light grey) or above (dark grey) normal range for Mountain Bay.

*Significant difference between sites (p ≤ 0.05) within years; ^a^ GSI = 100* (gonad weight/body weight); ^b^ LSI = 100* (liver weight/body weight); ^c^ K = 100*(weight/length^3^); ^d^ Represents an interaction between sites; All data before 2008 published in Bowron et al. (35).

During the gonadal recrudescent period (fall), white sucker were collected from Jackfish Bay (exposure) and Mountain Bay (reference) using overnight gill nets (8.9 cm and 10.2 cm mesh size, 150 m length) (**Figure 1**). Fish were removed from nets, placed in 200 L tanks containing fresh lake water, and returned to shore to be sampled immediately. Similar to spring sampling periods, 20 male and 20 female fish were targeted from each site following protocols described in the EEM program (52), with the exceptions of 1988 (50–60 fish of each sex), and 1990 (50–75 fish of each sex) (**Tables 5** **,** **6**).

**Table 5:** Age, length, weight, condition factor (K), gonadosomatic index (GSI) and liver somatic index (LSI) of female white sucker (*Catostomus commersoni*) from Jackfish Bay and Mountain Bay, collected during early-recrudescence (late summer/early fall) from 1988 to 2019.

Site	Date	Age (year)	Length (cm)	Weight (g)	GSI^a^	LSI^b^	K^c^	Fecundity
JFB	1988	9.3 ± 0.4 (56)*	40.6 ± 0.3 (56)*	1043.6 ± 20.0 (56)*			1.56 ± 0.01 (56)*	
	1989	10.7 ± 0.6 (15)*	40.6 ± 0.5 (15)	1112.8 ± 36.7 (15)	2.23 ± 0.13 (15)	3.07 ± 0.15 (15)*	1.66 ± 0.03 (15)*	
	1990	10.7 ± 0.3 (75)*	41.2 ± 0.2 (75)*	1022.6 ± 16.2 (75)	4.38 ± 0.24 (57)	2.15 ± 0.08 (52)*	1.46 ± 0.02 (75)*	
	1991	10.9 ± 0.6 (26)	41.5 ± 0.6 (26)*	1081.7 ± 44.8 (26)	3.32 ± 0.20 (26)*	1.74 ± 0.13 (16)*	1.50 ± 0.03 (26)*	
	1992	11.1 ± 0.5 (19)*	41.9 ± 0.6 (20)*	1117.8 ± 45.5 (20)	3.86 ± 0.22 (20)	2.78 ± 0.27 (16)*	1.52 ± 0.04 (20)*	
	1993		41.0 ± 0.5 (15)*	1005.5 ± 32.9 (15)*	4.03 ± 0.15 (15)*	2.02 ± 0.17 (15)*	1.47 ± 0.02 (15)*	
	1995		40.5 ± 0.7 (27)	1007.0 ± 51.5 (27)	4.54 ± 0.22 (27)	1.93 ± 0.10 (27)*	1.49 ± 0.03 (27)*	
	C1995	7.5 ± 0.5 (20)*	38.8 ± 1.1 (20)*	967.5 ± 86.5 (20)	4.43 ± 0.54 (20)	2.03 ± 0.09 (20)	1.57 ± 0.03 (20)*	
	C1999	7.3 ± 0.5 (24)*	38.7 ± 0.6 (24)*	959.6 ± 56.0 (24)*	4.42 ± 0.27 (21)	1.61 ± 0.06 (24)	1.63 ± 0.05 (24)*	31451 ± 2563 (21)
	2000	9.2 ± 0.4 (19)*	42.1 ± 0.6 (20)*	1221.5 ± 58.0 (20)	5.70 ± 0.38 (20)	1.86 ± 0.04 (20)	1.62 ± 0.02 (20)*	31822 ± 364 (20)
	C2002	6.2 ± 0.4 (25)	40.7 ± 0.9 (25)	1001.4 ± 59.9 (25)	4.24 ± 0.22 (25)*	1.63 ± 0.06 (25)	1.45 ± 0.02 (25)*	
	2006	10.3 ±0.8 (21)*	43.3 ± 0.7 (21)*	1252.0 ±56.2 (21)	2.54 ± 0.11 (21)*	1.52 ± 0.09 (21)	1.54 ± 0.05 (21)*	33098 ± 2603 (20)
	2008	5.8 ± 0.4 (20)*	41.1 ± 0.7 (20)*	1142.5 ± 54.3 (20)	2.40 ± 0.25 (20)^d^	2.06 ± 0.09 (20)^d^	1.63 ± 0.02 (20)*	24268 ± 1879 (20)
	2011	8.2 ± 0.6 (18)	43.6 ± 0.7 (20)	1284.3 ± 65.7 (20)	3.46 ± 0.20 (20)^d^	1.48 ± 0.06 (20)	1.53 ± 0.03 (20)^d^	
	2012	10.0 ± 0.5 (25)	43.8 ± 0.6 (26)	1286.1 ± 44.3 (26)*	3.46 ± 0.14 (26)	1.63 ± 0.06 (26)	1.52 ± 0.03 (26)*	
	2013	7.4 ± 0.7 (20)*	43.2 ± 0.8 (20)	1267.5 ± 70.6 (20)*	2.49 ± 0.13 (20)	1.60 ± 0.09 (20)	1.55 ± 0.03 (20)^d^	
	2018	10.3 ± 0.5 (19)	44.4 ± 0.6 (20)	1328.0 ± 48.2 (20)*	3.13 ± 0.14 (20)	1.58 ± 0.10 (20)	1.51 ± 0.02 (20)*	38889 ± 2504 (19)*
	2019	7.9 ± 0.4 (20)*	43.8 ± 0.7 (21)*	1257.1 ± 59.1 (21)	2.57 ± 0.16 (21)*	1.47 ± 0.06 (21)	1.48 ± 0.03 (21)*	36024 ± 3559 (19)
MTB	1988	8.3 ± 0.4 (49)	43.2 ± 0.4 (49)	1129.2 ± 21.0 (49)			1.40 ± 0.02 (49)	
	1989	7.9 ± 1.0 (14)	41.7 ± 0.9 (14)	1122.1 ± 64.7 (14)	2.38 ± 0.12 (14)	1.35 ± 0.08 (14)	1.54 ± 0.03 (14)	
	1990	9.9 ± 0.4 (50)	42.4 ± 0.3 (50)	1031.1 ± 19.4 (50)	5.18 ± 0.36 (50)	1.74 ± 0.05 (49)	1.35 ± 0.01 (50)	
	1991	10.8 ± 0.7 (24)	44.4 ± 0.5 (24)	1135.1 ± 30.7 (24)	4.07 ± 0.11 (24)	1.40 ± 0.03 (24)	1.29 ± 0.02 (24)	
	1992	9.8 ± 0.3 (20)	43.4 ± 0.5 (20)	1077.8 ± 30.4 (20)	4.19 ± 0.14 (20)	1.55 ± 0.10 (11)	1.32 ± 0.02 (20)	
	1993		44.3 ± 0.3 (31)	1191.0 ± 20.0 (30)	5.34 ± 0.11 (29)	1.71 ± 0.05 (30)	1.30 ± 0.02 (30)	
	1995		42.4 ± 0.8 (18)	993.8 ± 50.3 (18)	4.97 ± 0.18 (18)	1.49 ± 0.05 (16)	1.30 ± 0.03 (18)	
	C1995	9.5 ± 0.6 (24)	42.4 ± 0.7 (24)	1049.0 ± 57.5 (24)	5.89 ± 0.32 (24)	2.00 ± 0.07 (23)	1.36 ± 0.03 (24)	
	C1999	10.1 ± 0.7 (21)	44.0 ± 0.5 (21)	1179.1 ± 38.6 (21)	5.53 ± 0.20 (21)	1.66 ± 0.05 (21)	1.38 ± 0.02 (21)	37069 ± 1878 (21)
	2000	12.0 ± 0.9 (21)	44.2 ± 0.7 (21)	1224.9 ± 42.4 (21)	6.74 ± 0.36 (21)	1.80 ± 0.11 (21)	1.41 ± 0.02 (21)	26739 ± 1749 (21)
	C2002	6.5 ± 0.4 (26)	42.1 ± 0.5 (26)	1027.3 ± 41.6 (26)	5.29 ± 0.19 (26)	1.63 ± 0.05 (26)	1.36 ± 0.02 (26)	
	2006	15.4 ± 1.1 (21)	45.7 ± 0.5 (21)	1242.6 ± 35.4 (21)	3.14 ± 0.12 (21)	1.36 ± 0.06 (21)	1.30 ± 0.02 (21)	36345 ± 3106 (18)
	2008	8.3 ± 0.9 (17)	44.4 ± 0.9 (19)	1215.6 ± 51.9 (19)	6.59 ± 0.23 (19)	1.86 ± 0.07 (19)	1.39 ± 0.03 (19)	23288 ± 1271 (19)
	2011	9.5 ± 0.7 (16)	44.3 ± 0.5 (20)	1163.4 ± 32.0 (20)	4.12 ± 0.10 (20)	1.49 ± 0.05 (20)	1.34 ± 0.02 (20)	
	2012	9.1 ± 0.7 (18)	42.3 ± 0.6 (18)	1004.2 ± 37.3 (18)	3.02 ± 0.09 (18)	1.33 ± 0.03 (18)	1.32 ± 0.02 (18)	
	2013	8.8 ± 0.3 (34)	44.1 ± 0.5 (34)	1118.5 ± 32.8 (34)	2.67 ± 0.11 (34)	1.58 ± 0.05 (34)	1.30 ± 0.02 (34)	
	2018	10.5 ± 0.5 (21)	44.6 ± 0.5 (21)	1146.5 ± 44.8 (21)	3.54 ± 0.19 (21)	1.37 ± 0.05 (21)	1.28 ± 0.02 (21)	29768 ± 1985 (14)
	2019	9.5 ± 0.3 (20)	46.4 ± 0.5 (20)	1266.0 ± 37.4 (20)	3.35 ± 0.22 (20)	1.49 ± 0.04 (20)	1.27 ± 0.03 (20)	34459 ± 1598 (19)

Values are reported as mean ± SE (n). Shaded cells are either below (light grey) or above (dark grey) normal range for Mountain Bay.

*Significant difference between sites (p ≤ 0.05) within years; ^a^ GSI = 100* (gonad weight/body weight); ^b^ LSI = 100* (liver weight/body weight); ^c^ K = 100*(weight/length^3^); ^d^ Represents an interaction between sites; All data before 2008 published in Bowron et al. (35).

**Table 6:** Age, length, weight, condition factor (K), gonadosomatic index (GSI) and liver somatic index (LSI) of male white sucker (*Catostomus commersoni*) from Jackfish Bay and Mountain Bay, collected during early-recrudescence (late summer/early fall) from 1988 to 2019.

Site	Date	Age (year)	Length (cm)	Weight (g)	GSI^a^	LSI^b^	K^c^
JFB	1988	9.0 ± 0.4 (41)*	39.4 ± 0.2 (41)*	964.5 ± 17.2 (41)*			1.59 ± 0.02 (41)*
	1989	12.3 ± 1.2(11)	39.5 ± 0.6 (11)*	1093.9 ± 47.4 (11)	2.89 ± 0.43 (11)*	2.30 ± 0.20 (11)*	1.78 ± 0.03 (11)*
	1990	9.5 ± 0.4 (50)*	39.1 ± 0.3 (53)*	924.4 ± 19.6 (53)	4.13 ± 0.18 (45)*	2.04 ± 0.13 (43)*	1.54 ± 0.02 (53)*
	1991	12.2 ± 1.0 (11)	39.2 ± 0.6 (11)*	950.9 ± 50.2 (11)*	4.98 ± 0.41 (11)*	1.92 ± 0.23 (11)*	1.56 ± 0.04 (11)*
	1992	9.8 ± 0.6 (28)	37.8 ± 0.6 (28)*	841.1 ± 42.3 (28)*	5.21 ± 0.35 (26)*	2.49 ± 0.20 (25)*	1.52 ± 0.02 (28)*
	1993		40.2 ± 0.5 (12)	983.6 ± 41.4 (12)	5.92 ± 0.40 (12)*	2.05 ± 0.22 (11)*	1.46 ± 0.02 (12)*
	1995		36.4 ± 0.4 (34)*	718.8 ± 27.4 (34)*	5.76 ± 0.28 (33)	1.41 ± 0.07 (22)*	1.48 ± 0.02 (34)*
	C1995	7.9 ± 0.5 (25)*	37.3 ± 0.8 (25)	803.0 ± 52.5 (25)	5.29 ± 0.37 (25)*	1.82 ± 0.10 (25)*	1.50 ± 0.03 (25)*
	C1999	6.2 ± 0.4 (26)*	37.1 ± 0.5 (26)*	837.7 ± 37.5 (26)*	6.25 ± 0.32 (24)	1.33 ± 0.05 (26)	1.61 ± 0.02 (26)*
	2000	8.0 ± 0.3 (20)*	39.1 ± 0.4 (20)*	960.5 ± 29.6 (20)*	4.76 ± 0.21 (20)*	1.54 ± 0.07 (20)*	1.61 ± 0.02 (20)*
	C2002	4.8 ± 0.4 (26)*	34.0 ± 0.7 (26)*	708.5 ± 47.1 (26)	6.02 ± 0.31 (26)	1.23 ± 0.06 (26)	1.47 ± 0.03 (26)*
	2006	9.6 ± 0.6 (20)*	40.6 ± 0.4 (20)	1047.3 ± 28.9 (20)*	3.73 ± 0.37 (20)	1.24 ± 0.06 (20)	1.57 ± 0.02 (20)*
	2008	6.3 ± 0.4 (18)*	39.8 ± 0.4 (20)*	1043.8 ± 33.6 (20)	5.55 ± 0.22 (20)	1.73 ± 0.11 (20)*	1.66 ± 0.03 (20)*
	2011	7.6 ± 0.6 (16)	40.3 ± 0.5 (20)	1056.7 ± 37.9 (20)	6.45 ± 0.33 (20)	1.23 ± 0.06 (20)	1.60 ± 0.02 (20)*
	2012	10.1 ± 0.5 (21)*	41.4 ± 0.4 (21)	1095.2 ± 37.0 (21)*	5.34 ± 0.41 (21)	1.28 ± 0.06 (21)^d^	1.54 ± 0.02 (21)*
	2013	7.2 ± 0.6 (20)	41.2 ± 0.6 (20)	1115.8 ± 49.2 (20)*	4.67 ± 0.41 (20)	1.40 ± 0.08 (20)	1.58 ± 0.04 (20)*
	2018	9.8 ± 0.6 (20)	42.0 ± 0.5 (20)*	1174.8 ± 37.2 (20)*	6.02 ± 0.31 (20)	1.29 ± 0.07 (20)	1.59 ± 0.03 (20)*
	2019	7.0 ± 0.3 (19)	40.8 ± 0.7 (19)	1039.3 ± 52.8 (19)	5.46 ± 0.44 (19)	1.33 ± 0.08 (19)	1.51 ± 0.02 (19)*
MTB	1988	10.7 ± 0.5 (51)	41.5 ± 0.3 (51)	1016.6 ± 15.1 (51)			1.42 ± 0.01 (51)
	1989	12.0 ± 1.0 (9)	42.5 ± 0.6 (9)	1180.7 ± 39.8 (9)	4.65 ± 0.36 (9)	1.17 ± 0.08 (8)	1.54 ± 0.04 (9)
	1990	12.5 ± 0.5 (49)	40.8 ± 0.2 (49)	924.5 ± 13.6 (49)	6.21 ± 0.13 (49)	1.36 ± 0.04 (46)	1.36 ± 0.02 (49)
	1991	10.5 ± 0.6 (22)	41.4 ± 0.4 (22)	975.9 ± 19.2 (22)	6.84 ± 0.25 (21)	1.11 ± 0.03 (22)	1.38 ± 0.03 (22)
	1992	9.7 ± 0.3 (19)	40.8 ± 0.4 (20)	953.7 ± 18.8 (20)	7.79 ± 0.29 (20)	1.25 ± 0.08 (8)	1.41 ± 0.03 (20)
	1993		41.4 ± 0.7 (15)	981.8 ± 27.2 (15)	7.81 ± 0.21 (15)	1.24 ± 0.06 (15)	1.38 ± 0.05 (15)
	1995		39.4 ± 0.7 (12)	833.3 ± 44.5 (12)	6.48 ± 0.33 (12)	1.10 ± 0.06 (12)	1.35 ± 0.02 (12)
	C1995	9.2 ± 0.6 (25)	39.6 ± 0.5 (25)	851.0 ± 33.4 (25)	6.70 ± 0.22 (25)	1.22 ± 0.04 (25)	1.34 ± 0.02 (25)
	C1999	9.7 ± 0.7 (24)	41.0 ± 0.6 (25)	971.2 ± 39.2 (25)	6.63 ± 0.23 (25)	1.27 ± 0.03 (25)	1.39 ± 0.02 (25)
	2000	12.5 ± 0.7 (19)	41.9 ± 0.3 (20)	1038.9 ± 22.3 (20)	5.71 ± 0.16 (20)	1.24 ± 0.04 (20)	1.41 ± 0.02 (20)
	C2002	6.2 ± 0.5 (26)	39.2 ± 0.6 (26)	820.4 ± 40.9 (26)	6.70 ± 0.22 (26)	1.22 ± 0.04 (26)	1.34 ± 0.02 (26)
	2006	13.3 ± 1.2 (20)	41.3 ± 0.4 (19)	936.5 ± 31.0 (19)	2.82 ± 0.29 (19)	1.25 ± 0.09 (19)	1.33 ± 0.03 (19)
	2008	9.5 ± 0.8 (18)	41.7 ± 0.4 (18)	1021.9 ± 28.8 (18)	5.75 ± 0.23 (18)	1.42 ± 0.05 (18)	1.40 ± 0.02 (18)
	2011	8.6 ± 0.6 (14)	41.2 ± 0.3 (19)	979.1 ± 20.1 (19)	6.46 ± 0.22 (19)	1.12 ± 0.04 (19)	1.40 ± 0.01 (19)
	2012	11.7 ± 0.3 (19)	41.6 ± 0.4 (19)	954.7 ± 31.6 (19)	6.05 ± 0.34 (19)	1.15 ± 0.04 (19)	1.32 ± 0.02 (19)
	2013	8.2 ± 0.2 (27)	41.4 ± 0.3 (27)	970.0 ± 23.7 (27)	4.82 ± 0.29 (27)	1.20 ± 0.03 (27)	1.36 ± 0.02 (27)
	2018	9.6 ± 0.5 (18)	39.6 ± 0.4 (18)	842.2 ± 26.0 (18)	6.68 ± 0.33 (18)	1.18 ± 0.06 (18)	1.35 ± 0.02 (18)
	2019	7.9 ± 0.3 (11)	41.2 ± 0.7 (11)	907.4 ± 45.8 (11)	6.07 ± 0.62 (11)	1.05 ± 0.05 (11)	1.29 ± 0.03 (11)

Values are reported as mean ± SE (n). Shaded cells are either below (light grey) or above (dark grey) normal range for Mountain Bay.

*Significant difference between sites (p ≤ 0.05) within years; ^a^ GSI = 100* (gonad weight/body weight); ^b^ LSI = 100* (liver weight/body weight); ^c^ K = 100 × (weight/length3); ^d^ Represents an interaction between sites; All data before 2008 published in Bowron et al. (35).

During all sampling periods, blood was collected from fish by caudal puncture using syringes (5 mL 21 gauge 1.5 in long - Becton Dickinson (BD) Mississauga, ON, CAN) placed into vacuum tubes (BD Vacutainers, with lithium [LH] heparin 68 USP units) and stored on ice for later centrifugation. Blood samples were centrifuged (IEC Centra CL-2; Thermo Scientific; Waltham, MA, USA) at 8000 rpm for 10 min to separate red blood cells from plasma, which was drawn off and stored at −80°C for future steroid analysis. Fork length (± 0.1 cm), whole body weight (± 0.1 g), liver and gonad weights (± 0.01 g) were measured and recorded. A subjective index for secondary sex characteristics (tubercle index) and for the amount of visceral lipid stores was recorded in most years, with 1 representing low levels and 5 representing very high levels (1). The left operculum was collected to determine age by counting annuli and focusing on the distinction between winter and summer growth zones (53). Additionally, a 1-g ovarian sample was collected to estimate fecundity (number of eggs per fish) and approximately 1 g of liver was collected and flash frozen in liquid nitrogen for later determination of mixed function oxygenase (MFO) activity using 2,5-diphenyloxazole (PPO) (1) or ethoxyresorufin-o-deethylase (EROD) activity (54). Additional liver tissue in certain years was collected and analyzed for PCDD/F concentrations (**Table 7**; 49).

**Table 7:** PCDD/F concentrations (pg∙g^−1^, mean ± SD) with wet weight (pg∙g^−1^ wet wt., mean ± SD) and lipid normalized (pg∙g^−1^ lipid, mean ± SD) in liver tissue of male white sucker collected from Jackfish Bay and Mountain Bay during the fall.

	Jackfish							Mountain				
	1989	1991	1993	1995	2011	2012	2018	1989	1991	2011	2012	2018
**2,3,7,8-TCDD**	44.2±19.6	52.4±17.5	17.7±4.83	2.70±1.14	2.79±1.72	1.37±0.98	0.65±0.28	4.03	ND	0.73±0.17	0.47±0.41	0.26±0.22
**1,2,3,7,8-PeCDD**	ND	2.12±0.64	6.38±6.19	ND	0.56±0.19	0.43±0.11	0.31±0.12	ND	ND	0.62±0.18	0.42±0.19	0.22±0.09
**1,2,3,4,7,8-HxCDD**	ND	0.43	3.44±3.30	ND	0.19±0.09	0.20±0.05	0.10±0.02	ND	ND	0.19	N.D	0.12±0.09
**1,2,3,6,7,8-HxCDD**	1.68	0.61±0.11	3.05±3.35	1.11	0.26±0.12	0.20±0.09	0.22±0.10	ND	16.5±19.0	0.35±0.07	0.27	0.14±0.08
**1,2,3,7,8,9-HxCDD**	ND	ND	3.03±3.59	ND	0.18	0.15±0.04	0.09±0.02	ND	ND	0.15	0.20	0.12±0.09
**1,2,3,4,6,7,8-HpCDD**	1.68	1.43±0.21	4.56±4.32	1.64±0.16	0.26±0.07	0.07	0.30±0.13	ND	3.77	0.30±0.02	0.23±0.12	0.20±0.10
**OCDD**	120±161	4.38±1.02	4.67±3.25	13.4±10.2	0.32±0.15	0.53±0.53	0.41±0.15	35.54	18.9±16.0	0.25±0.07	0.44±0.07	0.28±0.20
**2,3,7,8-TCDF**	285±136	265±111	101±49.1	21.0±9.32	33.6±23.9	20.8±17.2	9.71±4.86	20.1±3.65	5.35±3.16	2.53±0.68	1.31±0.67	1.05±0.43
**1,2,3,7,8-PeCDF**	ND	3.68±1.20	5.34±4.04	2.15±1.83	0.39±0.17	0.33±0.15	0.22±0.13	ND	ND	0.35±0.09	0.20±0.10	0.19±0.21
**2,3,4,7,8-PeCDF**	10.4±3.98	12.5±2.84	5.09	ND	1.33±0.69	0.75±0.31	0.59±0.30	ND	1.55	0.59±0.15	0.46±0.26	0.28±0.17
**1,2,3,4,7,8-HxCDF**	ND	0.72±0.16	2.75±3.21	1.06	ND	0.15±0.06	0.07±0.02	ND	ND	N.D	N.D	0.11±0.08
**1,2,3,6,7,8-HxCDF**	ND	0.29	2.53±3.11	ND	ND	0.20	0.06±0.01	ND	ND	N.D	N.D	0.11±0.08
**1,2,3,7,8,9-HxCDF**	ND	ND	2.60±3.11	ND	ND	0.16±0.06	0.06±0.01	ND	ND	N.D	N.D	0.11±0.08
**2,3,4,6,7,8-HxCDF**	ND	0.32	2.49±3.05	ND	ND	0.16±0.05	0.06±0.01	ND	ND	N.D	N.D	0.11±0.08
**1,2,3,4,6,7,8-HpCDF**	ND	1.49±0.19	4.07±4.21	ND	ND	0.15±0.03	0.10±0.03	ND	ND	N.D	0.22	0.11±0.07
**OCDF**	ND	4.63±0.49	2.85±3.11	ND	ND	0.21±0.10	0.13±0.04	ND	ND	N.D	0.28	0.19±0.11
**TOTAL TETRA-DIOXINS**	44.4±19.5	52.4±17.5	17.7±4.83	2.76±1.16	3.04±2.04	1.49±1.15	0.56±0.34	6.59±3.61	ND	1.73±1.03	0.33	0.45±0.28
**TOTAL PENTA-DIOXINS**	ND	2.12±0.64	6.38±6.19	ND	0.57±0.19	0.41±0.13	0.20±0.20	ND	ND	0.62±0.18	0.29	0.19±0.12
**TOTAL HEXA-DIOXINS**	1.68	0.91±0.37	8.37±9.97	1.11	0.30±0.20	0.29±0.18	0.20±0.15	ND	12.0±15.6	0.83	0.47	0.12±0.09
**TOTAL HEPTA-DIOXINS**	2.31	1.68±0.52	4.90±4.02	1.64±0.16	0.28±0.06	0.19±0.04	0.23±0.20	ND	3.77	0.30±0.02	0.14	0.14±0.12
**TOTAL TETRA-FURANS**	285±136	270±107.7	101±49.1	21.1±9.32	34.2±24.2	22.6±18.3	9.96±5.06	20.1±3.65	6.32±3.57	4.84±1.66	3.41±3.74	1.19±0.41
**TOTAL PENTA-FURANS**	10.8±4.22	16.8±4.09	8.33±4.15	2.15±1.83	2.10±1.32	1.33±0.73	0.78±0.66	ND	1.55	2.58±1.70	1.89±1.80	0.54±0.26
**TOTAL HEXA-FURANS**	ND	1.17±0.28	10.4±12.5	1.06	ND	0.47±0.50	0.07±0.02	ND	ND	0.32	N.D	0.11±0.09
**TOTAL HEPTA-FURANS**	ND	1.49±0.19	4.07±4.21	0.64	ND	0.28±0.12	0.08±0.03	ND	ND	N.D	0.22	0.11±0.07
**N**	4	5^a^	4	8	6	6	18	3	3	3	3	18
**% lipid**	17.8±8.80	14.8±4.38	67.1±12.4	7.21±2.63	18.9±5.17	11.5±5.49	12.9±4.7	17.9±10.6	17.7±4.15	22.4±12.2	8.59±2.96	8.75±2.44
**TEQ^b^**	65.6±28.0	74.3±20.9	29.1±11.6	4.82±1.78	5.82±3.42	3.34±2.05	2.35±1.07	5.44±1.89	1.93±0.85	1.88±0.45	1.06±0.69	0.85±0.53
**TEQ_(lipid)_^b^**	416±225	563±199	42.4±9.42	70.8±27.1	30.2±13.2	27.8±6.24	18.5±6.23	33.3±8.14	11.9±6.84	9.44±3.06	11.8±3.85	9.47±4.36

ND, not detected; ^a^ Lipid normalized TEQ values calculated using n=4; ^b^ TEQ calculated using fish TEFs reported by Van den Berg et al. (55).

All sex steroids were measured *via* radioimmunoassay (1989 – 2013) or ELISA (2018–2019) following McMaster et al. (56). Stored plasma samples were extracted with ethyl ether to remove steroids, reconstituted in 1.0 mL of phosgel, and stored at −20°C. Radioimmunoassay or ELISA were run to determine the amount of 11-ketotestosterone (KT; males only), 17β estradiol (E2; females only), and testosterone (T; both sexes) using the methods described in Van Der Kraak et al. (57), Van Der Kraak and Chang (58), and Wade and Van Der Kraak (59), respectively.

For simplicity of presentation, summary tables and figures show ratio data for gonadosomatic index (GSI), liver somatic index (LSI), and condition factor (K). GSI is defined as the percent of whole body weight that is gonad, LSI is defined as a percent of whole body weight that is liver, and K is defined as a ratio between the fish’s body weight and length (K = 100 × weight/length^3^).

### Population Modeling

Population estimates were modeled for white sucker at Jackfish Bay over the time period of 1988 through 2019 using a density dependent population projection model (8, 60–62). The model uniquely combines a Leslie population projection matrix (63, 64) and a discrete time form of the logistic equation (65, 66) to translate changes in the vital rates and the age structure of a population of white sucker exposed to pulp mill effluent to alterations in population growth rate over time.

### Data Analysis

Data were analyzed for variability over time at the reference site, and across years for differences between sites. To focus on meaningful change, the Canadian EEM program uses critical effect sizes to identify changes that warrant further attention at exposed sites: 10% for changes in fish condition factor and 25% for all other endpoints relative to reference (52, 67). Due to the extended nature of this study, environmentally relevant changes are interpreted as those outside of “normal ranges” (68) which demonstrate when levels at Jackfish Bay were outside of expected normal ranges defined by 30 years of data at the reference site. In situations where values changed significantly over time at the reference site, normal ranges were broken down by study Phase described below.

All data were analyzed using SigmaPlot or Systat (Systat Software Inc., San Jose, CA, USA) unless otherwise specified. Males and females were analyzed separately and data were log transformed when necessary. Site differences for mean fork length, whole body weight, and fish age were determined using 1-factor analysis of variance (ANOVA) followed by a Tukey’s *post hoc*. Analysis of covariance (ANCOVA) followed by a Tukey’s *post hoc* was used to compare gonad weight and liver weight using body weight as a covariate or to compare body weight using length as a covariate between sites. Size at age, comparing mean fork length or mean weight between sites using age as a covariate was also analyzed by ANCOVA, when a significant relationship between these parameters was shown by regression analysis. When regression lines did not describe the data significantly, data were analyzed to see if the covariate was significantly different between sites; if not, data for the parameter of interest were compared by ANOVA. If there was a significant interaction in the ANCOVA (and regression lines were significantly different), the analysis was stopped, and the difference in the slope of the relationship was interpreted as being a significant difference between sites in the relationships. If the interaction was found not significant, the interaction term was removed and the analysis was re-run to determine whether there was a significant difference in intercepts between sites. Tests of statistical significance were set at an alpha (α) value of 0.05.

**Figure 2:**
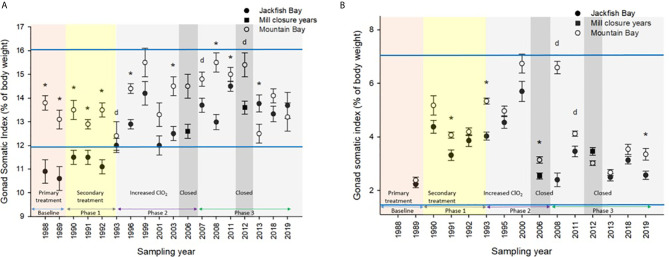
Gonad somatic indices (± SE) of female white sucker (*Catostomus commersoni*) from Jackfish Bay and Mountain Bay, **(A)** pre-spawning (spring) and **(B)** early-recrudescence (fall) from 1988 to 2019. Asterisks* represent a significant difference (p ≤ 0.05) between sites within years, “d” represents an interaction between sites, horizontal blue bars represent the 30 year normal range for the reference site. All data before 2008 published in Bowron et al. (35).

White Sucker data collected from 1988 to 2019 at Mountain Bay were used to generate normal ranges (69) for specific health endpoints (i.e. condition, GSI, etc.) by taking the grand mean ± 2 SDs, which encapsulate 95% of the observations from an assumed normal distribution. For example, the “normal range” for female GSI at the Little Gravel River Mountain Bay reference site during the spring spawning period was calculated to be 13.9 ± 2.02 (range from 1988 to 2019) (**Figure 2**). Similar calculations were conducted for other endpoints in the two seasons and were used to evaluate when Jackfish Bay fish were outside of normal.

## Results

Data were separated into four time periods for analyses; baseline (primary effluent treatment 1988–1989), Phase 1 (post-secondary treatment from 1990 to 1993) representing the early years of monitoring, Phase 2 (1996–2007) representing the period after modernization of bleaching process at the mill, and Phase 3 (2008–2019) representing the most recent sampling period and data which has not been previously published. Data are also displayed in periods relating to major treatment improvements, process changes and closures (e.g., **Table 2**).

### Baseline (1988–1989)

**Figure 3:**
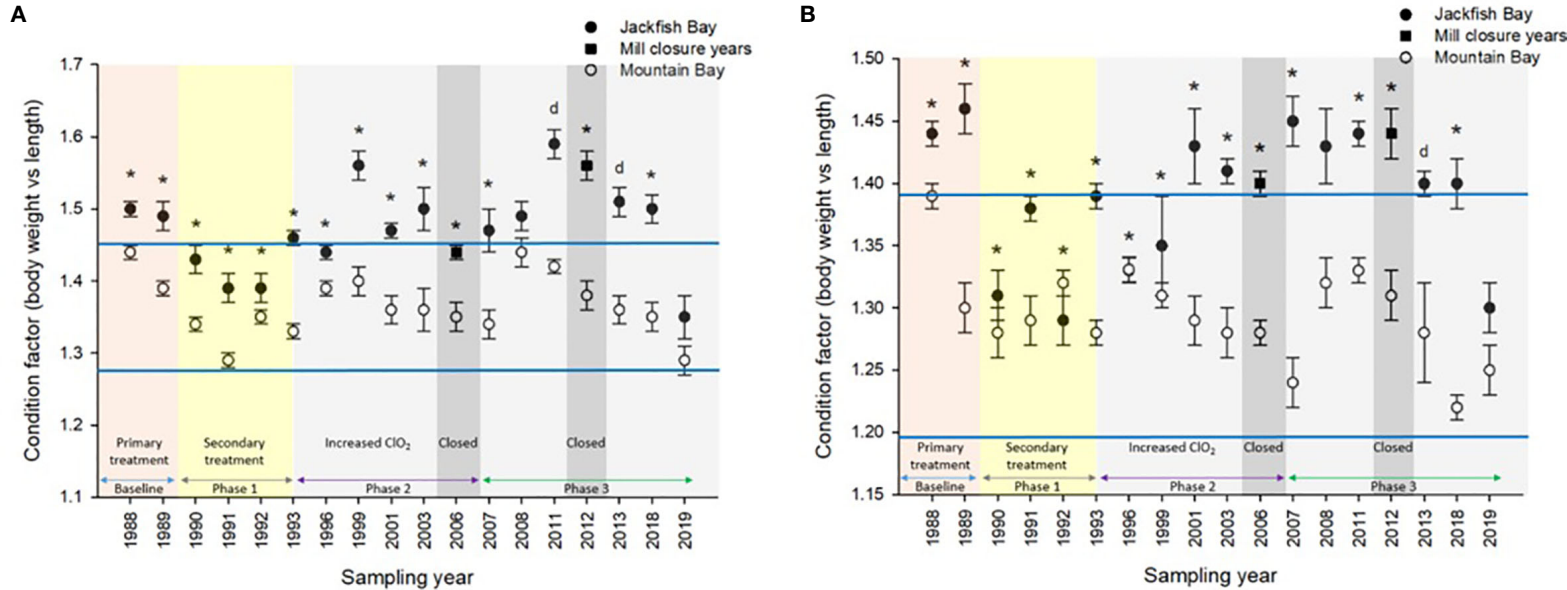
Condition (± SE) of **(A)** female and **(B)** male white sucker (*Catostomus commersoni*) from Jackfish Bay and Mountain Bay, pre-spawning (spring) from 1988 to 2019. Asterisks* represent a significant difference (p ≤ 0.05) between sites within years, “d” represents an interaction between sites, horizontal blue bars represent the 30 year normal range for the reference site. All data before 2008 published in Bowron et al. (35).

**Figure 4:**
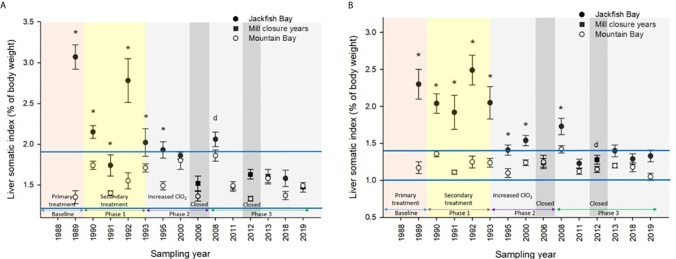
Liver somatic indices (± SE) of **(A)** female and **(B)** male white sucker (*Catostomus commersoni*) from Jackfish Bay and Mountain Bay, during the fall from 1988 to 2019. Asterisks* represent a significant difference (p ≤ 0.05) between sites within years, “d” represents an interaction between sites, horizontal blue bars represent the 30 year normal range for the reference site. All data before 2008 published in Bowron et al. (35).

**Figure 5:**
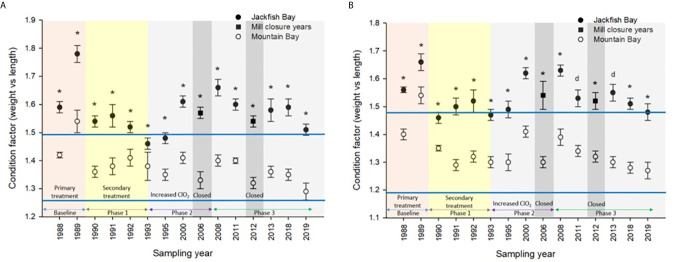
Condition (± SE) of **(A)** female and **(B)** male white sucker (*Catostomus commersoni*) from Jackfish Bay and Mountain Bay, during the fall from 1988 to 2019. Asterisks* represent a significant difference (p ≤ 0.05) between sites within years, “d” represents an interaction between sites. All data before 2008 published in Bowron et al. (35).

During the baseline years of primary treatment (1988 and 1989), both female and male white sucker sampled from Jackfish Bay during the spring (prespawning period) were older, shorter, and had higher condition factors than white sucker from Mountain Bay (**Tables 3**, **4** and **Figure 3**). Additionally, Jackfish Bay females were lighter, and had smaller relative liver sizes in 1989 (**Table 3**). In fall 1988 (early recrudescent period), female and male fish sampled from Jackfish Bay were shorter, weighed less, and had higher condition factors, with older females and younger males found at the exposed site (**Tables 5**, **6** and **Figure 5**). In fall 1989, exposed female white sucker were again older, had larger relative livers, and higher condition factors, while males were shorter, larger relative livers, and higher condition factors compared to unexposed fish (**Tables 5**, **6** and **Figures 4**, **5**). During the baseline period prior to secondary treatment, LSI and condition were outside the normal range for the reference site for males in prespawning and fall sampling periods, and for females for condition in both periods, and liver size in the fall sampling period (**Tables 3** **–** **6**). PCDD/F levels in livers were elevated more than 10-fold (**Table 7**), and liver MFO enzyme activity levels were significantly elevated in fall, although less so in the prespawning period (**Table 8**).

**Table 8:** Values at Jackfish Bay in different phases of the study (reported as % of Mountain Bay).

Season	Sex	Phase		Age (year)	Length (cm)	Weight (g)	GSI^a^	LSI^b^	K^c^	Fecundity	Maturity (%<7)	Fat	Tubercles	T	E2/KT	EROD	Dioxin (TEQ)
PRES	F	Baseline		123.8	96.1	96.2	80.0	92.1	105.7	91.7	34.6	97.8		35	92	159	
		1		132.4	96.3	97.0	88.3	108.0	106.8	106.8	41.1	38.5		29.3	185	365	
		2		87.6	94.5	91.5	89.5	91.0	108.3	111.9	142.8	74.5		74.6	150	95	
		3		94.1	100.6	112.9	96.2	96.9	109.2	142.3	89.6	54.2		61.1	202.0	141	
																	
PRES	M	Baseline		116.8	96.2	91.8	87.2	89.1	108.0		48.8	76.4	51.6	36.5	46.4	215	
		1		109.8	95.4	90.5	85.8	92.3	103.9		22.1	52.3	59.7	50.3	57.4	315	
		2		77.9	94.7	93.7	88.3	79.2	108.4		720.9	90.5	105.5	67.5	75	153	
		3		93.7	99.5	109.2	94.4	87.6	109.1		124.7	94.6	88.7	93.2	88.7	203	
																	
Fall	F	Baseline		123.7	95.7	95.8	93.7	227.4	107.8			80		45	26.6	728	
		1		107.4	94.9	95.7	83.4	136.3	113.2			51.7		80.9	39	2097	
		2		81.0	93.6	95.5	82.0	107.2	114.7					78.2	79.9	267	
		3		88.6	97.7	110.1	82.2	108.0	116.7			108.5		991.1	58.1	534	
																	
Fall	M	Baseline		93.3	93.9	93.8	62.2	247.0	115.6			88		36.7	29.1	1576	1123
		1		97.7	95.1	96.5	70.5	171.9	110.0			64.7		56.7	31.7	1055	2679
		2		78.3	92.6	92.9	94.6	117.7	113.2					79.4	74.1	538	252*
		3		86.6	99.6	115.6	93.6	115.9	116.8			132.7		70.7	56.2	376	330

Gray shaded cells represent values decreasing toward reference (light grey) or increasing toward reference (dark grey) and shaded boxes with white text represent increasing away from reference.

*Phase 2 Mountain Bay PCDD/F missing; reference average of 1991[1.93] and 2001[1.88].

During the baseline period, male GSI was outside the normal range of reference fish in prespawning and fall sampling periods (**Tables 4**, **6**), and female gonad size was below the normal range in prespawning (**Figure 2**), while fecundity was significantly reduced in females and male secondary sex characteristics were diminished (tubercle index was <50% of normal) (**Table 8**). Age at maturity was estimated by the proportion of spawning fish <7 years of age; Jackfish Bay white sucker showed fewer young fish in the spawning run in both females (34% of reference) and males (49%) (**Table 8**). Testosterone (T) levels were <40% of reference in both males and females during prespawning, and <50% of reference during the fall (**Table 8**). E2 and KT levels were <30% of reference in the fall in females and males, respectively, and while prespawning KT was <50% of normal, E2 levels were >90% of normal (**Table 8**).

### Phase 1 (1990–1993)

During Phase 1 (1990–1993), some endpoints showed partial recovery toward reference levels (normal range) after the installation of secondary treatment, with an improvement in liver size in the fall (falling from 227% of normal to 135% of normal in females; males were unchanged at 190% of normal), although it was still significantly different and outside normal range for the reference site (**Figure 4**). Although prespawning female condition and liver size were statistically different 7 of 8 times, they were only outside of normal range once (**Table 3**). For pre-spawning males, condition remained statistically higher in each year although it was only outside of normal range once (**Table 4**). Condition factor in male fish showed a marginal improvement in the fall, (113% of normal to 110% of normal), although condition factor was the most consistently impacted endpoint across all years (**Table 6** and **Figure 5**). For fall males, liver size and condition factor were significantly elevated and outside normal range 7 of 8 sampling periods, and fall female condition factor and liver size remained statistically elevated in all years and outside of normal range six of eight times (**Table 5** and **Figure 5**). PCDD/F and MFO levels remained elevated in males in the fall (**Table 8**), and MFOs were higher in both sexes during prespawning and in females during the fall (**Table 8**).

Prespawning female gonad size remained below normal range and male gonad size remained lower and outside of normal range 3 of 4 sampling years (**Tables 3**, **4** and **Figure 2**). No significant difference in fecundity was observed during Phase 1 (1990–1993) of the monitoring study, although fecundity was only 83% of normal during the first year of baseline sampling (**Table 8**). Fall male gonad size was reduced significantly in all years while female gonad size was only significantly smaller in 2 of 4 years; in both cases GSIs remained within the normal range of reference values (**Tables 5**, **6** and **Figure 2**). Prespawning T levels in females and age-at-maturity remained low at Jackfish Bay, but were slightly improved in males, although tubercles (secondary sex characteristics) and KT remained low (**Table 8**). Fecundity in Jackfish Bay females recovered to reference levels and prespawning E2 was elevated. Fall E2 and KT remained <40% of reference levels although T levels showed some recovery in both males and females (**Table 8**).

### Phase 2 (1995–2007)

After significant changes in process and bleaching sequences at the mill, (Phase 2; 1995 – 2007), some endpoints continued to show improvement/recovery, moving toward reference fish levels especially during the prespawning sampling period. The average liver size in females was actually smaller than reference sites (92% of reference), and in males averaged only 79% of reference levels and was outside of normal range in 3 of 6 years during the prespawning period (**Table 4**). Condition factor in fish collected during the spring remained unchanged in both males and females at just over 108% of reference (**Tables 3**, **4** and **Figure 3**). Liver size was reduced from previous years at the exposure site during the fall and was 117% of normal in females (down from 135% in Phase 1) and 106% of reference levels in males (**Figure 4**), although condition factor increased to 113% of reference in females and 115% in males (**Tables 5**, **6** and **Figure 5**). PCDD/F levels (**Table 7**) and MFO activity (**Table 8**) were markedly reduced, although they remained elevated above reference.

Prespawning gonad size improved marginally in females to 89% of reference from 85% in previous years (**Figure 2**), and prespawning male gonads were only significantly smaller in the first year of sampling in Phase 2, averaging 106% of reference levels (**Tables 3**, **4**). Fecundity at Jackfish Bay increased to an average of 112% of reference levels and was significantly higher twice. Age at maturity and tubercle indices returned to levels above reference, and hormone levels were 75% or more of reference except for male prespawning T (68%) and female E2 remained elevated (150%) (**Table 8**). Fall gonad size remained reduced and was 91% of reference in males and averaged 81% of normal in females (**Figure 2**), although differences were not always statistically significant and remained within normal ranges (**Tables 5**, **6**).

### Phase 3 (2008–2019)

In the most recent years of sampling (Phase 3, 2008-present), some endpoints continued to show improvement/recovery with exposed fish endpoints moving toward reference fish levels especially during the prespawning period. Similar to Phase 2, prespawning liver size remained smaller at Jackfish, averaging 95% of reference levels in females and 87% in males. Condition factor in both males and females remained 109% of normal (**Tables 3**, **4**and **Figure 3**). Liver size continued to improve in fall collections and was down to 108% of normal in females (down from 117% in Phase 2 and 135% in Phase 1), although male liver size increased to 117% of reference (up from 106% in Phase 2) (**Tables 5**, **6** and **Figure 4**). Condition factor was slightly elevated from Phase 2, with both males and females in the fall being in excess of 116% of reference levels (**Figure 5**).

Prespawning gonad size improved further in females to 95% of normal from 85% in previous years, and prespawning male gonads averaged 94% of reference during Phase 3 (**Tables 3**, **4** and **Figure 2**). Fecundity at Jackfish continued to increase, averaging 140% of reference fecundity levels. Prespawning male hormone levels were about 90% of normal, while female levels remained altered (reduced T and elevated E2). Female fall T levels returned to normal, while E2 and male T and KT remained reduced at about 60–70% of normal (**Table 8**). Fall differences remained significant, with fall gonad size averaging 75% of reference levels in females (**Figure 2**) and 93% of reference in males; although differences tended to be interactions (**Tables 5**, **6** **)**. PCDD/F levels continued to decrease in Phase 3 from 2011 to 2018 but were still significantly higher than the reference site (**Table 7**).

**Figure 6:**
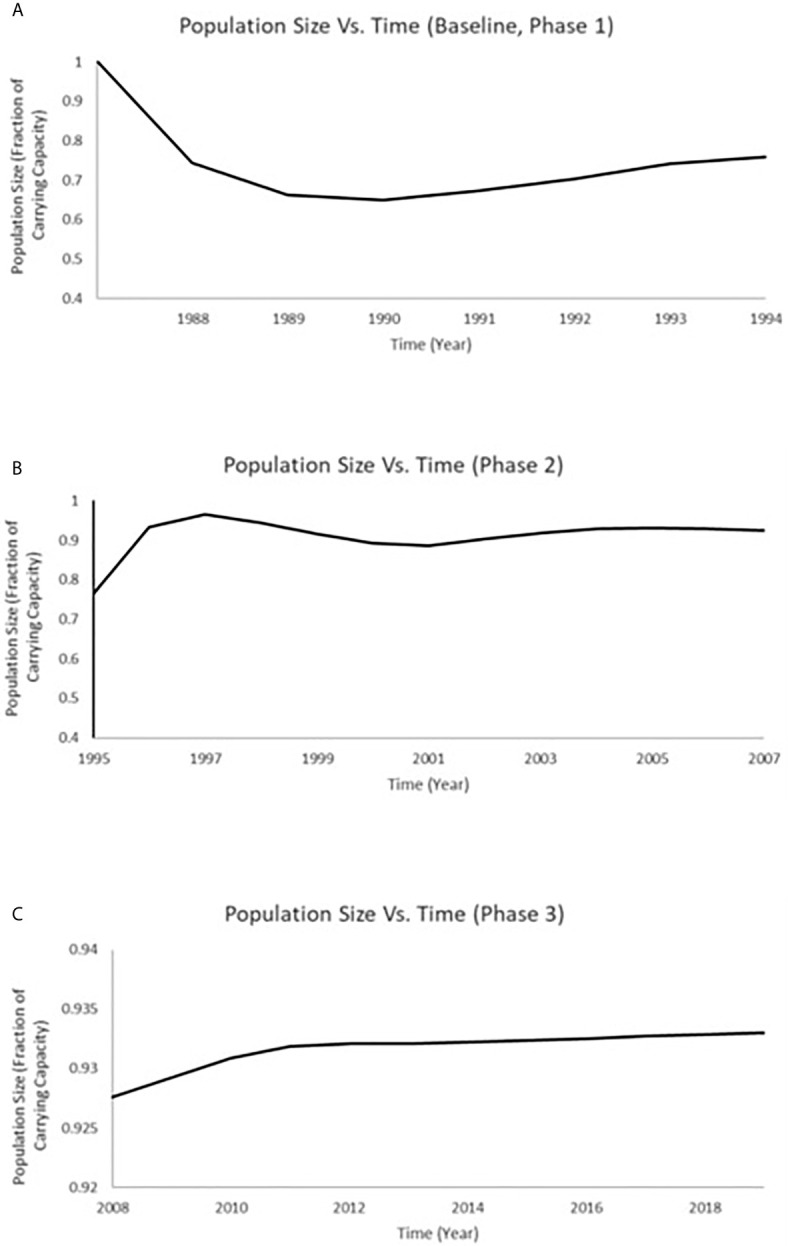
Time series of total population size for white sucker at JFB that includes the timeframe of 1988 through 2019. **(A)** The predictions for total population size at JFB from Miller et al. (8) for the years 1988 to 1995 (which includes both baseline and Phase 1); **(B)** The predictions for total population size at JFB modeled for 1996–2007 (Phase 2); **(C)** The predictions for total population at JFB modeled for 2008 to 2018 (Phase 3).

Population models were estimated by considering the measurements of endpoints for population recovery recorded in connection with the changes that took place during both the Phase 2 (1996–2007) and Phase 3 (2008–2019) periods. A time series of total population size was projected for white sucker at Jackfish Bay that includes the timeframe of 1988 through 2019 (**Figure 6**). We used the predictions for total population size at Jackfish Bay from Miller et al. (8) for the years 1988 to 1995 (which includes both baseline and Phase 1; **Figure 6**). In determining population size projections for the 1995 to 2007 (Phase 2) and 2008 to 2019 (Phase 3) time periods, we compared the relative difference between values from the Jackfish Bay and Mountain Bay sites (**Table 3**) to adjust both the fecundity and age of breeders accordingly in the population model for each annual time step. The population at Jackfish Bay was expected to recover to above 90% of carrying capacity by the end of Phase 2 (**Figure 6**) and to further approach recovery to near 93.5% of carrying capacity by the end of Phase 3 (**Figure 6**).

## Discussion

The Jackfish Bay studies represent a unique long-term examination of biological responses in fish to major changes in the pulp mill industry, including installation of waste treatment, process changes, and both maintenance and temporary mill shutdowns. The response pattern seen in white sucker during the baseline period (1989) demonstrated clear endocrine disruption with reductions in circulating and *in vitro* production of sex steroids, gonad size, fecundity and secondary sex characteristics. Detailed studies showed an impact on the sensitivity of the ovulatory response to gonadotropin injections, an impaired pituitary response and reductions in *in vitro* steroid synthesis (5). Studies on *in vitro* steroid synthetic capacity identified a number of alterations in the steroid biosynthetic pathway adding to the reproductive effects (6), although the major alteration appeared to be due to reductions in the availability of the steroid substrate cholesterol (7). Further studies provide evidence that pulp and paper mill effluent impairs expression of reproduction-related genes (70), and contain neuroactive substances that interact with several neurotransmitter receptors (such as the dopamine, gamma-aminobutyric acid, glutamate, and acetylcholine receptors) and enzymes important in regulating reproduction (71). Effluents have also been shown to have both estrogenic (72, 73) and androgenic effects (74).

The 30-year period of Jackfish Bay studies and the sequencing of mill changes allow a closer examination of the benefits of technology upgrades and the relative importance of endocrine impacts in terms of consequences to whole organism responses. Secondary treatment was installed at the pulp mill in October 1989 (after the 1989 fall baseline collections) and consisted of an aerated stabilization basin with a 10-day retention time. Studies conducted between 1990 and 1993 after these treatment additions (Phase 1) demonstrated small improvements in gonad size (8% in prespawning females and fall males), and fecundity returned to reference levels. The biggest improvement was seen in fall liver size; a 75% improvement in females and a 50% improvement in males. Fall MFO activity showed a 50% improvement in males (less induction) but significantly higher induction levels were found in females. Secondary sex characteristics remained low and age to maturity remained delayed, and all sex hormone levels remained depressed. Similar delays in maturity have been observed in wild fish sampled near a Swedish pulp mill in the Bothnian Sea (24), in mummichog (*Fundulus heteroclitus*) in the Miramachi Estuary (New Brunswick, Canada) exposed to bleached kraft mill effluent (BKME; 75), and in fathead minnow (*Pimephales promelas*) exposed in the laboratory to a variety of BKMEs (23). Miller et al. (8), modeled these reductions in gonad size and fecundity under the exposure experienced during the period 1988 to 1994 in Jackfish Bay (Baseline and Phase 1), and estimated a 34% to 51% annual decrease in recruitment during that period of exposure which would then approach an equilibrium population size of 71% of the overall carrying capacity in the system.

The failure of biological endpoints in fish to recover after the installation of secondary treatment in 1990, and the confirmation of similar biological changes at other pulp mills, including some not using chlorine bleaching (4), led to the inclusion of a requirement for EEM in the Canadian 1992 Pulp and Paper Effluent Regulations, as well as the development of EEM programs in other countries including Chile (76) and Brazil (77). EEM is an industry-funded cyclical monitoring program to test whether a mill in compliance with its effluent limits is associated with impacts on fish (adult fish survey), fish habitat (benthic macroinvertebrate survey) or use of fish (PCDD/F survey) (78). EEM requirements have subsequently been developed under Fisheries Act regulatory changes for metal mines, coal mines, diamond mines and are under consideration for other sectors. For pulp and paper, EEM operates on a 3-year cycle and analysis of studies from cycle 2 (2000) and cycle 3 (2003) concluded that the average national response pattern in fish was increased liver size and condition factor and reduced gonad size (13–15), mimicking the early Jackfish Bay studies.

Metabolic disruption in wild fish has been documented in mills utilizing a variety of different pulp production and effluent treatment types, characterized by increased body condition, increased liver size, and decreased gonad size relative to reference sites (11). Similar to Jackfish Bay white sucker, Baltic perch (*Perca fluviatilis*) exposed to BKME near Norrsundet, Sweden displayed fast growth and a high condition factor that was accompanied with a significant delay in sexual maturation (79). Additionally, early evidence of changes in secondary sex characteristics induced by exposure to pulp mill effluent was observed by the masculinization of female mosquito fish (*Gambusia affinis holbrooki*) in the Fenholloway River of Florida (80, 81). Although installation of secondary treatment at the Terrace Bay mill resulted in some improvements in the metabolic disruption response pattern seen in Jackfish Bay white sucker, effects were still observed including age to maturation and expression of secondary sex characteristics.

In the fall, white sucker showed increased liver sizes, increased condition factor, but reductions in qualitative visceral lipid index and in gonad size (1). Fish condition was consistently elevated throughout the studies in both sexes at Jackfish Bay but increased over time in females during both prespawning and fall collection periods. Elevated condition factor suggests sufficient food was available but was not converted into reproductive investments (e.g. reduced GSI). Similar to results from other early pulp mill studies, these changes were interpreted as a form of nutrient enrichment combined with metabolic disruption as fish exposed to mill effluent had disrupted ability to allocate available resources to reproduction (2).

Reduction in gonad size relative to the reference site has been consistently measured in white sucker exposed to BKME in Jackfish Bay since the late 1980s (35). This depression in gonad size has been seen in wild fish exposed to BKME in Sweden (79, 82), Finland (83), and New Zealand (84), and was associated with a concomitant delay in age to maturation of fish in Canada (2) and Sweden (79). Further evidence of reproductive impacts have been seen in wild fish at other mills in Canada, USA (reviewed in 25) and Brazil (85), and in a variety of laboratory studies (28) demonstrating the potential of pulp and paper mill effluents.

A series of process changes took place at the mill between 1993 and 1998, including a concentrator addition for the recovery boiler, two effect concentrators to increase liquor solids, replacement of old wooden stave piping, a change in the chlorine generator with a gradual increase to 100% Elemental Chlorine Free (ECF) bleaching, and the addition of hydrogen peroxide to the bleaching process. The process changes implemented widely at Canadian mills during this time were in response to new 1992 Federal regulations under the Canadian Environmental Protection Act limiting the release of polychlorinated dibenzo-*p*-dioxins and furans (PCDD/F). In Phase 2 studies (1995–2007), there was a rapid improvement in PCDD/F levels in fish liver and a 50% improvement in liver MFO activity. Studies during a temporary mill maintenance shutdown in 1991 strongly suggested that the compounds responsible for MFO induction were not PCDD/F, as hepatic MFO levels returned to normal after a 7-d mill maintenance shutdown (19). Changes in the mill to eliminate molecular chlorine resulted in the total PCDD/F in fish measured in 2012 to be only 3.34 ± 2.05 (ng/g TEQ), roughly 21 times lower than was measured 21 to 23 years earlier (**Table 7**), and this was similar in the PCDD/F record in surface sediments in cores (49).

Phase 2 fall fish studies demonstrated that there were large improvements in hormones, including male T and KT, and continued improvements in gonad and liver size (35). During the prespawning period, there were improvements in female T and male T and KT but levels still remained more than 25% depressed relative to reference levels. There was also a dramatic improvement in the proportion of young fish and in the spawning run, recovery of secondary sex characteristics, continued increase in fecundity. Miller et al. (62), used a simple density-dependent logistic matrix model linked to adverse outcome pathways for reproductive effects (reduced T) and showed that process changes at the mill corresponded to improvements in population status. Advanced maturation was also observed in brook stickleback (*Culaea inconstans*) sampled from Moberly Lake between 2007 and 2013 (86). In studies at other mills, improved reproductive performance was generally associated with reduced use of molecular chlorine, improved condensate handling and liquor spill control (25) and at mills with lower BOD and gas chromatographic total ion profiles (28, 87). A study of female white sucker in the Androscoggin River, Maine, USA found that the pattern of reproductive impacts observed near 3 pulp mills disappeared after a switch to ECF bleaching, including reductions in gonad size and plasma E2 and increase in plasma T in fish exposed to effluent (88).

It is clear that despite the recovery of some endpoints after mills have switched to ECF effluents, constituents in mill effluent are still capable of affecting steroid hormone production. Karels et al. (89) continued to see a significant decrease in E2, T and vitellogenin gene expression in female roach (*Rutilis rutilis*) with an increase of E2 and decrease in T in male roach exposed to BKME in Lake Saimaa, Finland after the mill switched to be ECF. Estrogenic effects were also observed in both laboratory and *in situ* rainbow trout caging studies conducted downstream of ECF pulp mills in Chile (72) and in juvenile rainbow trout exposed to mill effluent extracts from Canada, Brazil and New Zealand exposed *via* intraperitoneal injections of a solid-phase extraction–dichloromethane nonpolar fraction (73). As well, both estrogenic and androgenic affects were observed in wild fish caught downstream of ECF mills in the Biobio River Basin in Chile (74).

The most recent Jackfish Bay studies (2008–2019; Phase 3) showed continued improvements in prespawning gonad sizes and fall liver sizes toward reference; although condition factor remained high, fecundity continued to increase (up to 142% of reference), and average age decreased (**Table 8**). Jackfish Bay white sucker currently display a population response pattern of improved food conditions (90) with improved reproduction (showing higher fecundity and younger average age of fish during spawning runs). During the prespawning sampling period, relative gonad size in females from Jackfish Bay steadily increased through the monitoring years toward reference fish gonad size, ending at 96.2% on average in Phase 3. Larger livers are not always observed in fish exposed to pulp and paper mill effluent (91), although larger livers are seen twice as often as reduced liver sizes near pulp mill effluent in Canada (92).

Population status for white sucker at Jackfish Bay over the full study period (1988 through 2019) was investigated using a density dependent population projection model (8, 60–62). The model uniquely combines a Leslie population projection matrix (63, 64) and a discrete time form of the logistic equation (65, 66) to translate changes in the vital rates (fertility and survivorship) and the age structure of a population of white sucker exposed to pulp mill effluent to alterations in population growth rate over time. Previous application of the model demonstrated that a white sucker population existing at carrying capacity and subsequently exposed to pulp mill effluent equivalent to the documented exposure experienced during the period 1988 to 1994 (baseline and Phase 1) in Jackfish Bay would be expected to exhibit an annual decrease in recruitment (ranging from 34% to 51% during the first 5 years of exposure) (8). The population was projected to approach an equilibrium population size of 71% of carrying capacity (8). With improvements in reproductive endpoints following process and treatment changes over the last two decades, we expanded upon that previous modeling by considering the measurements of endpoints for population recovery during both the Phase 2 (1996–2007) and Phase 3 (2008–2019) periods. The population at Jackfish was expected to recover to above 90% of carrying capacity by the end of Phase 2 and to further approach recovery to near 93.5% of carrying capacity by the end of Phase 3. This modeling approach can be further employed to project recovery as more data becomes available in moving beyond the 2019 sampling period at this Great Lakes Area of Concern in Recovery.

Mill closures showed strong evidence that complete recovery is possible and that current discharges are still exerting some effects. A temporary mill shutdown for maintenance in 1991 was associated with a recovery in liver MFO activity (19). During the spring of the first mill closure in 2006, female endpoints from Jackfish fell within the normal range of reference fish for all endpoints in white sucker. Although not always significant, females and males from Jackfish Bay continued to be younger, shorter, and weighed less than females from the reference site after the mill closure in February 2006. During the second mill closure in 2012, Jackfish Bay white sucker showed similar improvements compared to the 2006 shutdown. Prespawning Jackfish Bay female and male fish were younger and shorter compared to reference fish. Although not always significant, females were heavier and males were lighter compared to reference fish. Females sampled during prespawning showed an interaction between sites in gonad size, with larger fish showing smaller gonads than the reference site, however no difference was observed in gonad size in male fish.

In 1987, the International Joint Commission identified 43 Areas of Concern (AOC) in the Great Lakes with 12 in Canada and 5 connecting channels (Rivers connecting the lakes) with the USA. The goal was to remove all identified Beneficial Use Impairments (BUIs) and to then delist those sites. In May of 2011, Jackfish Bay was designated as an “AOC in recovery”, meaning that all remedial actions are complete and monitoring is underway to track the natural restoration of beneficial uses (https://binational.net/annexes/a1/). Although fish health specifically was not identified as a BUI, these studies provide input to the recovery process in Jackfish Bay. For example, gonadosomatic indices were smaller and outside of reference normal 11 times prior to 2011 and only once for male white sucker during the spring of 2018 demonstrating significant recovery in gonadal effects at Jackfish Bay. Similarly, fall liver size has demonstrated a significant improvement over the years with only males in the fall of 2013 having liver sizes outside of reference normal as demonstrated in **Figure 4**. Condition factor however has not demonstrated similar recovery as fish remain significantly fatter at the Jackfish Bay exposed location. Modeling of populations above also indicate a return to close to normal population size given increased fecundity and some recovery in gonad size. McMaster et al. (1) documented reduced egg size in females from Jackfish Bay and although percent hatch and survival were similar to the Mountain Bay reference, larvae were shorter at day 21 post hatch (56). Egg size was not measured in later studies, however given the increased fecundity and similar GSIs, egg size may likely still be reduced at Jackfish Bay.

Differences in species sensitivities to pulp and paper mill effluent have been observed throughout the years. Our early studies at Jackfish Bay demonstrated reproductive dysfunction in white sucker, longnose sucker (*Catostomus catostomus*) and lake whitefish populations exposed to BKME. Although all three species show elevated levels of MFO activity and depressed circulating steroid levels, only white sucker and lake whitefish showed impacts of reduced steroid levels on reproductive performance (93). Gibbons et al. (94) found that trout-perch (*Percopsis omiscomaycus*) and white sucker from a Kapuskasing mill exhibited in many ways, opposite responses to pulp mill effluents. Exposed trout-perch were shorter, lighter, younger, and had no difference in fish condition, GSI, or LSI compared to reference fish while exposed white sucker were longer, heavier, older, had increased condition, decreased GSI, and increased LSI relative to reference fish (94). Only exposed male trout-perch had elevated EROD activity and increased basal *in vitro* production of T by testicular tissue (94). During the development of laboratory tools for use in Investigation of Cause of the reproductive effects, fathead minnow 5 and 21 d, mummichog 25 d, and zebrafish (*Danio rerio*) 7 d tests, all of which had egg production as the primary reproductive endpoint were used. Additional bioassays examining reproductive-endocrine endpoints included a 7-d mummichog test, a 7-d and a 21-d three-spine stickleback (*Gasterosteus aculeatus*) test, a rainbow trout (*Oncorhynchus mykiss*) 7 d test, and *in vitro* sex steroid receptor and plasma protein binding bioassays. Different levels of species sensitivity were demonstrated with all effluents tested, and the fathead minnow 5 d egg production test was selected as the most sensitive to the effluents tested (26). Fathead minnow life cycle studies had also been shown to mirror effects demonstrated in the field when tested (95) supporting their responsiveness to effluent exposure.

The main impacts of concern in white sucker in the early years are no longer detectable or are much reduced; secondary sex characteristics, delayed maturity, and gonad sizes showed substantial improvements in prespawning males and females and fall males. The contamination of fish with PCDD/F has declined and is now essentially the same as reference sites. However, there are still significant differences in circulating hormone levels. With the exception of prespawning T levels in females, all steroids showed substantial improvement over the studies, but fall levels of E2 in females and T and KT in males remained below 80% of normal in both Phase 2 and 3, but well above the 26% to 46% of normal seen during the early studies. It is clear that fish can still reproduce successfully with some reductions in circulating hormone levels, and in fact, fecundity is 140% of reference in recent years. The reduced levels of gonadotropin in prespawning fish documented by Van Der Kraak et al. (5) would have been expected to have resulted in complete reproductive failure. However, McMaster et al. (56) collected spawning fish at both sites, eggs had matured naturally and fertilization studies concluded gametes of both sexes were viable. The decline in mean age of the spawning run at Jackfish Bay also supports the interpretation (90) that there are improvements in reproduction.

Increased E2 at prespawning is paradoxical. According to Van Der Kraak et al. (5) and evidence from *in vitro* studies (6), higher levels of E2 in spring correspond to altered control of reproductive function. Environmental cues result in a change in the steroid biosynthetic pathway away from T and E2 to 17α,20β-dihydroprogesterone the maturation inducing steroid. High levels of E2 just confirm the altered steroidogenic enzyme activities.

Pulp and paper mill effluents are complex matrices containing an array of causative compounds with each mill having a different complex mixture, making it difficult to generalize effects across pulp mills. Effluents contain materials extracted from wood (e.g., extractives, phytosterols, and trace metals), process derivatives/compounds formed during pulping/bleaching (e.g., dimethyl disulfide formed during kraft pulping), additives (e.g., polymeric formulations used as retention aids in papermaking), and biodegradation products of the aforementioned compounds if the effluent is biotreated (25). There are also complexities across mills in how black liquor is handled, what happens to condensates, and the types and efficiency of waste treatment. Furthermore, mills use different processes, different wood furnish, produce different products, and may change wood and products over the course of the year depending on demand. Much of the chemical characterization of mill effluents was performed during the 1980s and 1990s (96–98), with no detailed reports characterizing effluents following numerous process modifications over the last decade (25).

Data from studies throughout the years and the national monitoring program in Canada show that effluent-related effects on fish are not necessarily related to the type of manufacturing process (pulping and bleaching) but that observed effects are likely from a common source such as wood furnishes or additives used by the mills (25). Data also suggests that reproductive effects are minimized by improving mill operations in terms of spill prevention and effluent treatment (25), which is also apparent through our 30-year monitoring study at Jackfish Bay. It is important to again note the role biotreatment has on effluent quality in terms of reproductive effects, particularly in the relationship of BOD. The addition/improvement of effluent biotreatment typically reduces BOD by 90% or more, which eliminated acute lethal toxicity (25) and improved reproductive responses in both laboratory and wild fish populations however this treatment did not eliminate these responses (reviewed in 4, 20, 28, 99).

## Conclusion

Effluent-exposed white sucker from Jackfish Bay showed a response pattern of reduced gonad sizes and circulating sex steroid levels, increased liver size and condition factor, older age and delayed maturity. Although differences were still evident after installation of secondary treatment, liver size differences from reference were reduced by more than 25% in males and 50% in females, gonad size differences from reference were now less than 10%, and significant reductions in the age differences between sites occurred. Process changes, including conversion to high levels of ECF bleaching in Phase 2, greatly reduced TCDD/F exposure, reduced the larger liver sizes back to reference levels in some years, reduced gonadal size differences during fall recrudescent periods (but not in females prespawning), reduced induction of mixed function oxygenase enzymes (EROD), and was associated with elimination of age differences between sites. Circulating sex steroid levels, prespawning female gonad sizes and condition factor did not respond to either process changes or secondary treatment, but both circulating sex steroids and gonad sizes showed recovery to normal levels after the mill closed for an extended period (8 months). It is evident that some modernized mills can still release chemicals capable of impacting reproductive development of fish in the receiving waters. Although there were improvements in reproductive performance following process changes (including conversion to ECF bleaching), the mill shutdown, lasting 8 months, clearly demonstrated the potential for further improvement. Modeling of this recovery suggests that the white sucker populations should return to 93.5% of carrying capacity. While there are definitely differences between species, sites, effluents and studies conducted at pulp mill sites, the overall results of this comprehensive long-term study are directly supported by the data syntheses from the Canadian EEM Program that reproductive impacts in fish can persist at some modernized mills. It will be interesting to follow EEM National data assessments to evaluate whether best management practices implemented by the industry to reduce BOD in effluents below 20 mg/L results in reduced reproductive effects.

## Data Availability Statement

The raw data supporting the conclusions of this article will be made available by the authors, without undue reservation.

## Ethics Statement

The animal study was reviewed and approved by National Animal Care Program Environment and Climate Change Canada.

## Author Contributions

EU: investigation, formal analysis, and writing. MM: conceptualization, methodology, investigation, review and editing, and funding acquisition. MS: investigation, review, and editing. DM: formal analysis, review, and editing. KM: conceptualization, methodology, investigation, review and editing, funding acquisition, and supervision. All authors contributed to the article and approved the submitted version.

## Funding

This study has been supported over the years by a wide variety of funding agencies, graduate students, and technicians. The current study benefited by funding from the Canada Research Chairs and NSERC Discovery grant program (KM, MS), Environment and Climate Change Canada’s Great Lakes Action Plan (MM) and the University of New Brunswick, Wilfrid Laurier University and the University of Calgary (KM) and the University of Waterloo (MS).

## Conflict of Interest

The authors declare that the research was conducted in the absence of any commercial or financial relationships that could be construed as a potential conflict of interest.
